# Reproductive Mode and the Evolution of Genome Size and Structure in *Caenorhabditis* Nematodes

**DOI:** 10.1371/journal.pgen.1005323

**Published:** 2015-06-26

**Authors:** Janna L. Fierst, John H. Willis, Cristel G. Thomas, Wei Wang, Rose M. Reynolds, Timothy E. Ahearne, Asher D. Cutter, Patrick C. Phillips

**Affiliations:** 1 Institute of Ecology and Evolution, University of Oregon, Eugene, Oregon, United States of America; 2 Department of Ecology and Evolutionary Biology and Centre for the Analysis of Genome Evolution and Function, University of Toronto, Ontario, Canada; University of Edinburgh, UNITED KINGDOM

## Abstract

The self-fertile nematode worms *Caenorhabditis elegans*, *C. briggsae*, and *C. tropicalis* evolved independently from outcrossing male-female ancestors and have genomes 20-40% smaller than closely related outcrossing relatives. This pattern of smaller genomes for selfing species and larger genomes for closely related outcrossing species is also seen in plants. We use comparative genomics, including the first high quality genome assembly for an outcrossing member of the genus (*C. remanei*) to test several hypotheses for the evolution of genome reduction under a change in mating system. Unlike plants, it does not appear that reductions in the number of repetitive elements, such as transposable elements, are an important contributor to the change in genome size. Instead, all functional genomic categories are lost in approximately equal proportions. Theory predicts that self-fertilization should equalize the effective population size, as well as the resulting effects of genetic drift, between the X chromosome and autosomes. Contrary to this, we find that the self-fertile *C. briggsae* and *C. elegans* have larger intergenic spaces and larger protein-coding genes on the X chromosome when compared to autosomes, while *C. remanei* actually has smaller introns on the X chromosome than either self-reproducing species. Rather than being driven by mutational biases and/or genetic drift caused by a reduction in effective population size under self reproduction, changes in genome size in this group of nematodes appear to be caused by genome-wide patterns of gene loss, most likely generated by genomic adaptation to self reproduction per se.

## Author Summary

Closely related species can vary widely in genome size, yet the genetic and evolutionary forces responsible for these differences are poorly understood. Among *Caenorhabditis* nematodes, self-fertilizing species have genomes 20–40% smaller than outcrossing species. Constructing a high quality *de novo* genome assembly in *C. remanei*, we find that this outcrossing species has many more protein coding genes than the self-fertilizing *Caenorhabditis*. Intergenic spaces are larger on the X chromosome and smaller on autosomes for both selfing and outcrossing *Caenorhabditis*, but protein-coding genes are larger on the X chromosome in the self-fertile *C. briggsae* and *C. elegans* and larger on autosomes in the outcrossing *C. remanei*. This contrasting pattern of contracting genomes and expanding genes is likely mediated by changes in the balance between genetic drift and natural selection accompanying the transition to self-fertilization.

## Introduction

Self reproduction increases the probability of homozygosity at single loci, reducing the effective size of the population by a factor of two [1–3]. At the level of whole genomes, the reduced probability that two loci will be heterozygous within a single individual greatly decreases the efficacy of recombination, thereby increasing linkage disequilibrium within the population [4]. Increased homozygosity can expose heretofore recessive mutations to natural selection, potentially accelerating the rate of evolution at those loci, while increased linkage disequilbrium makes self-reproducing (“selfing”) species more susceptible to selective sweeps and/or background selection generated by advantageous and deleterious mutations [5]. What then should be the genomic consequences of a transition in mating system from outcrossing to selfing? The reduction in population size means that genetic drift should become more prominent, allowing for the accumulation of slightly deleterious features, such as repetitive elements, which should lead to an increase in genome size [6]. Similarly, increased linkage disequilibrium means that new advantageous mutations are more likely to bring along deleterious elements via hitchhiking as they increase in frequency [7].

Alternatively, any systematic mutational bias in the direction of DNA deletion would have a greater chance of succeeding within selfing species if such deletions are mildly deleterious [8], and even more so if the transition to selfing means that certain biological functions related to outcrossing (such as mate finding) are no longer needed. Further, increased linkage within selfing lineages increases the probability of co-inheritance of the host genome and selfish genetic elements such as transposable elements, which should lead to an increase in the efficacy of selection against the selfish elements and therefore a reduction in genome size if such elements are a significant fraction of the original ancestral genome [9]. Finally, in species with sex chromosomes, selfing has the potential to equalize the effective population size of sex chromosomes, which tend to have an *N*_*e*_ that is 3/4 as large as autosomes because of the reduced chromosome count in the heterogametic sex [1]. Although this is not usually an issue in plants, for which there are few species with sex chromosomes [10], in animals this change in the ratio of effective population size, as well as other sex-chromosome specific effects such as the lack of dominance in the heterogametic sex, could influence the rate of molecular evolution on sex chromosomes [11] following a transition to selfing. Thus, shifts in mating systems could potentially lead to either increases or decreases in genome size depending on the functional role and genomic context of a given segment of DNA. Determining what actually occurs in nature therefore depends both on a well-documented evolutionary transition in mating system and a set of well-annotated genomes that allow genetic function to be appropriately classified and compared.

Nematodes in the genus *Caenorhabditis* have made the transition from outcrossing to selfing three separate times independently [12, 13]. The well known model system *C. elegans*, as well as the species *C. briggsae* and *C. tropicalis*, reproduce primarily through self fertile hermaphrodites, which are essentially sperm-producing females derived from male-female (gonochoristic) ancestors. Males capable of mating with the hermaphrodites are also present at low frequencies within these species, but importantly, hermaphrodites are incapable of mating with each other. The genome sizes in *Caenorhabditis* nematodes are smaller by 20–40% for self-fertile hermaphrodites (*C. elegans*, 100.4Mb; *C. briggsae*, 108Mb; *C. tropicalis*, 79Mb) than the flow-cytometry estimated genome sizes of the larger *N*_*e*_ outcrossers (*C. remanei*, 131Mb; *C. brenneri*, 135Mb; *C. japonica*, 135Mb)[14–16]. A similar pattern of genome reduction has been observed in multiple self-reproducing plant species as well [9], which raises the possibility that genome size reduction may be a general syndrome associated with the transition to self reproduction.

Here, we combine existing genome assemblies with new functional annotation for each of these species in order to examine common features of genomic evolution that are shared across the three transitions in mating system from outcrossing to selfing within this genus. However, outcrossing nematode genome assemblies remain problematic because of remarkably high levels of DNA sequence variation in these species, including some of the most polymorphic animals currently known [17, 18]. This extreme polymorphism presents a particularly unique problem for genome assembly because the worm’s small size precludes sequencing a single animal, and so DNA must be extracted from populations that tend to remain highly polymorphic despite laboratory inbreeding [19]. Thus, in order to address questions about changes in finer-scale genomic structure under the transition to selfing, we also present a high-quality draft genome sequence for the outcrossing *C. remanei* and use this sequence to test theoretical predictions regarding the influence of self-fertilization on genome evolution. The combination of general comparisons across the phylogeny with specific comparisons between *C. elegans*, *C. briggsae*, and *C. remanei* shows that, while these nematodes share the general pattern of genome size reduction with plants, they appear to achieve it in different ways and that the particulars of the changes likely result from an interaction between genomic architecture and changes in population size and the frequency of interactions between the sexes.

## Results and Discussion

### Genomic comparisons

We analyzed the genome content of all *Caenorhabditis* members of the Elegans supergroup with genome sequences available on Wormbase [20]: the self-fertile hermaphrodites *C. briggsae*, *C. elegans*, and *C. tropicalis* and the outcrossing *C. remanei*, *C. japonica*, *C. brenneri*, and *C. sinica* (formerly *C*. sp. 5 [21]). In addition, we analyzed the outcrossing *C. angaria* as an outgroup [22]. We performed each analysis on both the extant *C. remanei* assembly and our new *de novo* assembly presented below. The results presented here are based on the *de novo* assembly, with the analysis of previously assembled (highly polymorphic) *C. remanei* genome sequence presented in the Supplemental Materials (S1 Table; S1 Fig). For each genome used here, we also generated a *de novo* functional annotation for each species using the same pipeline so as to minimize annotation bias from influencing the results (S2 Table).

**Fig 1:**
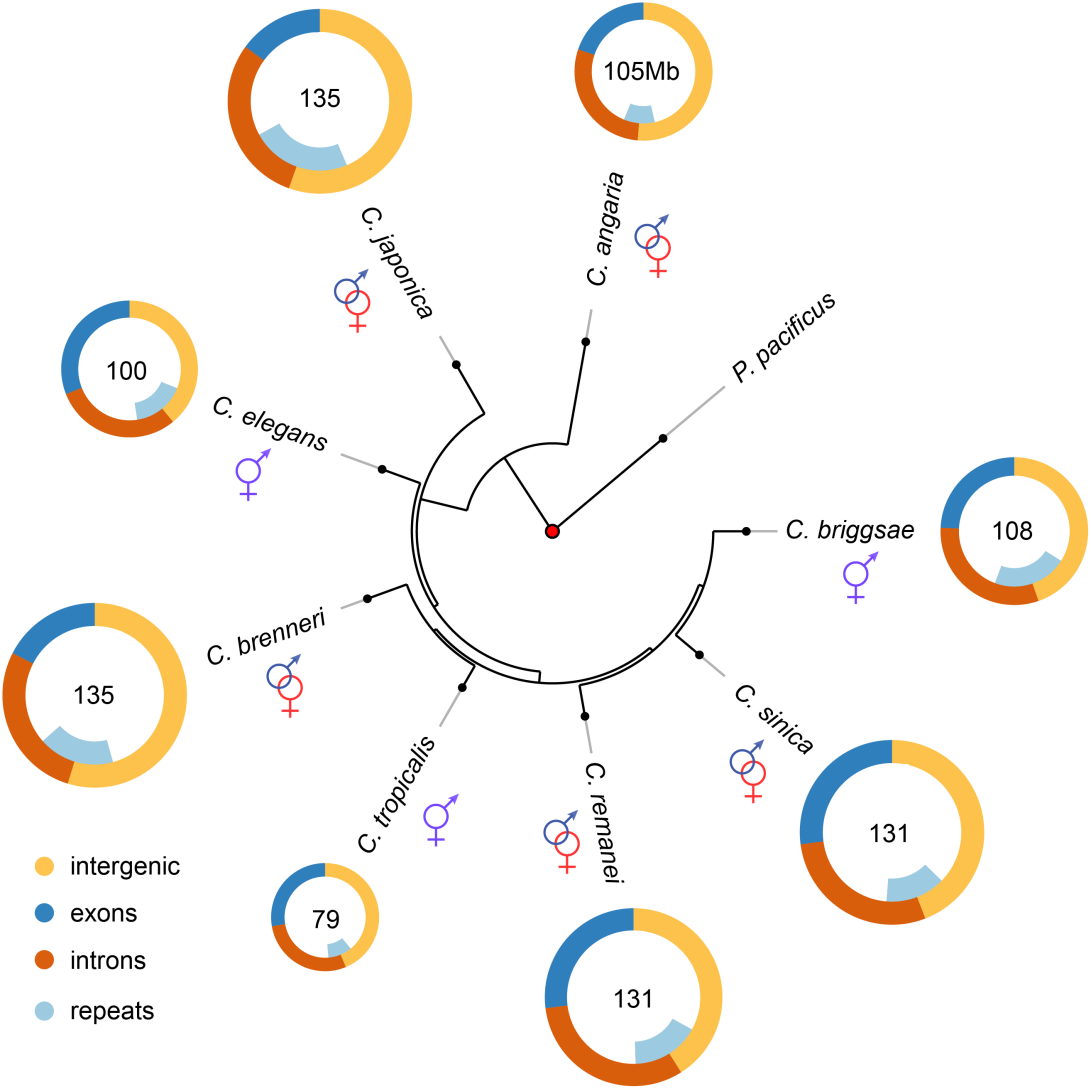
Genome content analysis across the *Caenorhabditis* Elegans supergroup (with outgroup species *C. angaria* and the distantly related *P. pacificus*). Genome content analysis does not support expansion of repeat elements in outcrossing species. The Elegans supergroup contains at least 17 species [93], but only the species whose genomes are analyzed here used are shown. The Elegans supergroup evolved from a common ancestor with a gonochoristic (*e.g*., male-female) mating system (identified with the red and blue symbols) and *C. japonica*, *C. brenneri*, *C. remanei* and *C. sinica* have retained the ancestral mating system. *C. elegans*, *C. briggsae*, and *C. tropicalis* have an androdioecious mating system with self-fertile hermaphrodites and males segregating at low levels in the populations.

Overall, consistent with previous genome size estimates based on flow cytometry [14, 15], self-fertile species within this group have substantially smaller genomes than their most closely related outcrossing relatives (Fig 1, Table 1, S2 Table). Taking the average genome size of the outcrossing species to be 130Mb, the genomes of *C. elegans*, *C. briggsae* and *C. tropicalis* have been reduced by 23%, 17% and 39% respectively via the transition to selfing. These are likely to be upper bounds on the actual size reduction because of likely over-assembly of most of the outcrossing species (see below). Although the branch lengths between each is actually fairly long (on the order of tens of millions of years), the actual time of the shift to selfing within any given lineage is likely to not have been more than ~4 million years based on the rate of evolution of codon usage bias [23]. To a first order, the overall pattern of genome shrinkage is roughly proportional when summed across broad functional categories, including the total size of exons, introns, intergenic regions, and repetitive elements within each genome (Fig 1; Table 1). Importantly, it does not appear that genome shrinkage is dominated by changes within a single functional class, such as repetitive elements. Thus, this comparative analysis suggests that genomic change following the transition to selfing is generated by a general reduction in genome size across coding and non-coding regions. A more precise analysis requires a careful comparison within each functional category, which in turn requires more complete genome assemblies for outcrossing species than are currently available within this group. To this end, we generated a *de novo* high quality assembly of *C. remanei* before completing the remainder of the tests.

**Table 1:** Summary of genomic characteristics.

		**Self-reproducing (androdioecious)**			**Outcrossing (gonochoristic/dioecious)**				
		*C. elegans*	*C. briggsae*	*C. tropicalis*	*C. remanei*	*C. sinica* ^1^	*C. brenneri* ^1^	*C. japonica* ^1^	*C. angaria*
Size of assembled genome (Mb)^2^	Exons^3^	33	27	23	30	39	37	27	23
	Introns	34	33	16	39	28	33	40	24
	Intergenic	39	49	39	50	64	120	99	59
	Total	100	108	79	119	131	190	166	105
Repetitive content	Coding^3^	9	11	4	9	10	7	14	4
	Noncoding	7	13	4	9	9	31	27	6
	Total	16	24	8	18	19	38	41	10
Estimated number of genes^3^		20,964	22,269	22,326	25,415	34,696	30,954	30,361	27,970

^1^Genome characteristics for these species are likely to be overestimates because of residual allelism within the genome sequences. Estimated actual genome size for *C. brenneri* and *C. japonica* is 135 Mb.

^2^Estimates for gene content and gene number for *C. elegans* and *C. briggsae* are slightly larger than their published values due to revision of the annotation and assembly since publication.

^3^Estimates for gene content and gene number as annotated in Wormbase release 244. The exonic, intronic, and intergenic contributions sum to greater than the total genome size because of alternative splicing.

### Assembly of the *C. remanei* genome

To create a reliable and well-assembled genome sequence for *C. remanei*, we first aimed to remove residual polymorphism from extant laboratory strains. We used a novel breeding scheme to create nearly isogenic strains for deep coverage genome sequencing, genetic mapping, and high-quality genome assembly. Specifically, we performed sequential inbreeding and selection over 50 generations to purge deleterious mutations and create 2 highly inbred *C. remanei* lines (New York PX356 and Ohio PX439). We then assembled a *de novo* draft genome sequence for PX356 from ~560x coverage of paired-end shotgun sequence, and ~75x coverage of 3 sizes of mate pair libraries. We used sequenced mRNA extracts from a mixed-stage population of *C. remanei* to annotate protein-coding genes.

**Fig 2:**
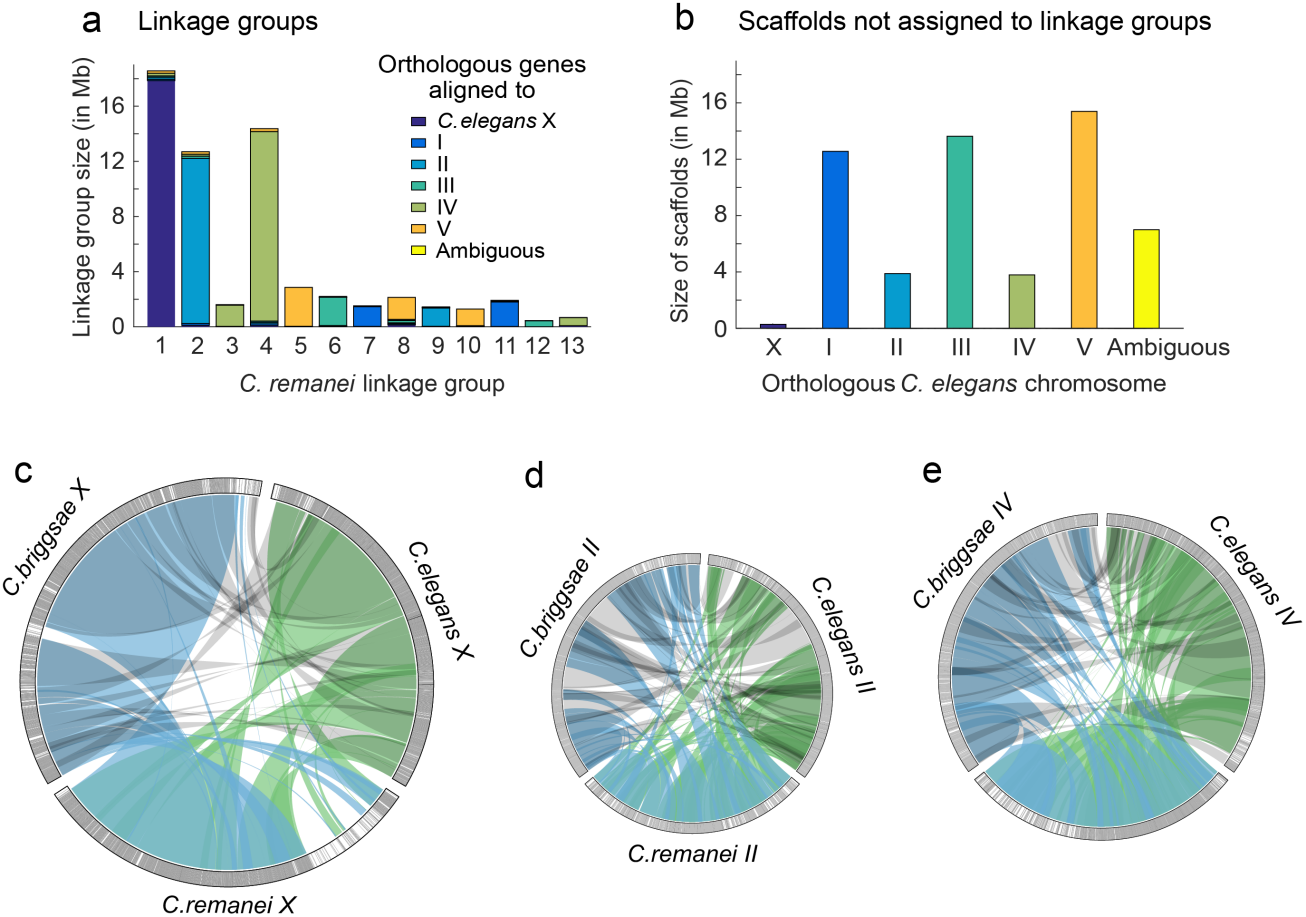
Whole chromosome comparisons among *C. elegans*, *C. briggsae*, and *C. remanei*. The *C. remanei* The linkage map was sufficient to assemble and order 98.93% of the scaffolds with orthologous genes aligning to *C. elegans* chromosome X, 78.38% of the scaffolds with orthologous genes aligning to *C. elegans* chromosome II and 81.40% of the scaffolds with orthologous genes aligning to *C. elegans* chromosome IV. (a) *C. remanei* linkage groups were assigned to chromosomes based on gene orthology to *C. elegans* chromosomes. Reproductive incompatibility between the *C. remanei* strains used to construct the linkage map resulted in over-dispersion of the linkage map and 13 linkage groups instead of the 6 chromosomes expected (both *C. elegans* and *C. briggsae* have 6 chromosomes, respectively). (b) The cumulative size and orthologous gene alignments for scaffolds that were not assigned to linkage groups. c-e) Orthologous gene alignments indicated blocks of syntenic DNA between *C. elegans*, *C. briggsae*, and *C. remanei*. The panels c-e show orthologous genes on chromosomes X, II, and IV, with chromosome size scaled to linkage group size in *C. remanei* (X 18.5Mb, II 12.5Mb, IV 14.5 Mb). Orthologous genes were connected between species pairs, and grouped together if the genes were within 50,000 nucleotides of each other. Single gene translocations were excluded for clarity. Green indicates orthologs identified between *C. elegans* and *C. remanei*, blue indicates orthologs identified between *C. remanei* and *C. briggsae*, and grey indicates orthologs identified between *C. briggsae* and *C. elegans*. The outer rings are chromosomes X, II, and IV in each species. Each gray line is an orthologous gene located on the same chromosome in the other species and each black line is an orthologous gene that is located on a different chromosome in one of the other species. There are few blocks of interchromosomal translocation, and few black lines. White indicates regions of the chromosome where there were no orthologous genes identified between the species. (c) There was a large region of divergence (roughly 3.6Mb) on the *C. remanei* X; (d) Chromosome II is not completely assembled in *C. remanei*, and there were several regions of *C. elegans* and *C. briggsae* chromosome II that were not represented in *C. remanei*; (e) Chromosome IV.

We estimated residual polymorphism in our inbred PX356 strain to occur at just ~0.01% of sites in well-assembled genic regions. In comparison, analyses of the previously assembled draft *C. remanei* genome [19] found allelic dimorphism for 4.7% of defined *C. elegans* orthologous genes and a sizable portion of DNA aligning to the *C. elegans* Chromosome IV (~10% of the total genome). Unfortunately, cryptic reproductive incompatibilities between PX356 and PX439 led to significant segregation distortion for several linkage groups in our genetic map. Overall, our assembly appears to provide good coverage for linkage groups orthologous to *C. elegans* Chromosomes II, IV, and X, with more fragmented coverage of Chromosomes I, III, and V (Fig 2). Nevertheless, gene assemblies within these large fragments are excellent. This therefore represents the first well-assembled genome from a highly polymorphic outcrossing species from this group. When looking at the evolution of genome structure we concentrate on the subset of well-assembled chromosomes, while when looking at the evolution of gene structure, we include the entire genome assembly.

### Evolution of transposable element copy number

We first tested the hypothesis that change in genome size in selfers is driven by a reduction in transposable element (TE) abundance [24]. TEs vary widely in structure, mobility, distribution, and diversity [25] and, depending on the dynamics of these factors and population size, transposons are predicted to increase genome size in selfers relative to outcrossers [26] or outcrossers relative to selfers [27].

Within this group of nematodes, the *C. briggsae* genome is 8Mb larger than the *C. elegans* genome largely owing to repeat content [28], with roughly 8% of the *C. briggsae* genome composed of Tc1-IS630-Pogo DNA transposons [29]. We found that the repeat content of *C. remanei* is intermediate between *C. elegans* and *C. briggsae* (Fig 1; Table 1; S2 Table), and therefore expansion of repetitive DNA within *C. remanei* can not explain genome size differences. The self-fertile *C. tropicalis* has the smallest genome (79Mb) of the Elegans supergroup, as well as the smallest repeat content. However, the self-fertile *C. briggsae* has a repeat content larger than any of the outcrossing *Caenorhabditis* with the exception of *C. japonica*. We therefore find no evidence that repeat expansion and/or shrinkage explains the majority of genome size differences between outcrossing and self-fertile *Caenorhabditis*, although it is a minor contributing factor for *C. elegans* and *C. tropicalis*. This is in stark contrast to plants, in which it appears that reduction in TE content is one of the major factors driving the evolution of smaller genome size within the self-compatible species [30].

### Biased insertion/deletion frequencies

Accumulated biases in insertion or deletion mutations may grow or shrink genomes across different scales. For example, Hu et al.[30] examined the basis for genome size differences between the self-fertile plant *Arabidopsis thaliana* (with a 125Mb genome) and the outcrossing *A. lyrata* (with a 207Mb genome), and found hundreds of thousands of small deletions in the self-fertile *A. thaliana*. Alternatively, insertions and deletions could occur at the level of individual genes; *A. thaliana* has 17% fewer genes than *A. lyrata*[30]. Thomas et al.[31] found that selfing *Caenorhabditis* have smaller transcriptomes than related outcrossers and a specific reduction in expression of genes that show sex-biased expression in outcrossing *Caenorhabditis*. Large-scale rearrangements may also account for genome size differences, and comparison between the *A. thaliana*-*A. lyrata* genomes discovered 3 large deletions in the selfing species [30].

Biases in the distribution of indels can be driven by selection or via neutral processes. Rapid growth and reproduction may favor small genomes [32], and self-fertile organisms with these life cycles [33] may experience selection for DNA loss. Parasites are expected to be more frequent in outcrossing populations than selfing, and theoretical studies indicate that parasites may select against gene loss in hosts [34]. Alternatively, neutral differences in mutational processes may result in genome size differences [35, 36]. Size transmission bias, whereby the XX hermaphrodites tend to inherit chromosomes shortened by deletions and XO males tend to inherit longer chromosomes with transgenic insertions, has been reported in *C. elegans*[37]. Although the mechanism through which this occurs is not yet known, simulations indicate that the androdioecious mating system of self-fertilizing *Caenorhabditis*, with males contributing few offspring to most generations, could rapidly lead to reduced genome size via this mechanism [37].

First, we explored the role of large-scale insertions and deletions contributing to genome size differences within *C. elegans*, *C. briggsae*, and *C. remanei*, as this analysis requires well-assembled chromosomes. These species have diverged from a common ancestor at least 30 million years ago [38], which means that few sequences other than those within conserved coding regions can be aligned between them. In order to align large genomic regions, we identified 15,699 orthologous genes among the three species, finding that >90% of these orthologous genes are found on the same chromosome in all three (Fig 2). The X chromosome in particular retains a striking conservation of synteny, with the exception of an apparent *C. remanei*-specific ~3.6Mb region of divergence (Fig 2C). This latter portion of the *C. remanei* X chromosome contains 786 annotated genes, but only 48 of these are orthologous to genes in *C. elegans* (47 to genes in *C. briggsae*), and of these, only 17 orthologous genes are found on the *C. elegans* X (the same 17 genes have orthologous counterparts on the *C. briggsae* X). Fifty of the genes in this region are orthologous to genes in *C. brenneri* but in the absence of a *C. brenneri* genetic map we do not know if these are located on the X chromosome.

Genes within a highly divergent syntenic region may retain similar biological functions despite a lack of clear orthologous counterparts. We used Interproscan v5.3 to assign putative biological functions to protein domains for the clusters of genes with no apparent syntenic relationships across species. For example, roughly 7% of the genes in the *C. elegans* genome are seven transmembrane G protein-coupled serpentine receptors (7TM GPCRs)[39]. These chemosensory genes are responsible for recognition of food, environment, and other animals, and thought to be found in large numbers in *Caenorhabditis* because of the soil/decaying-plant-dwelling nematodes need to respond to environmental cues [39]. There are 19 families of serpentine receptors in *C. elegans*. The majority of members within a family occur as clusters on chromosome arms, thought to be the result of local gene duplication events [40]. The *C. remanei* genome contains 59 *serpentine receptor class g* (*srg*) genes. Six *srg* genes (10.17% of the total) are located in this region of the X chromosome (2.74% of the total genome), and the genes surrounding each of these *srg* genes show no functional or sequence similarity to the genes surrounding the 72 *C. elegans* *srg* genes or the 60 *C. briggsae* *srg* genes. Thus, this region of the X appears to largely hold *C. remanei*-specific genes and to not be translocated relative to other chromosomes in other species.

We next tested the accumulated insertion/deletion hypothesis for genome size change by focusing on indel size biases in smaller blocks of aligned sequence. Consistent with the larger-scale comparisons, we found no difference in indel size bias among species when we analyzed individual syntenic blocks of DNA between species pairs (S2 Fig). In particular, there is no evidence that these aligned regions tend to be systematically smaller in *C. elegans* and *C. briggsae* relative to *C. remanei*. Although there are a few specific differences among species, overall we do not find evidence that either large-scale rearrangements or small-scale indels are an important contributor to genome size differences among these species.

### Protein-coding genes

Our *de novo* *C. remanei* genome assembly predicts a complement of 25,415 protein-coding genes, compared to 20,532 in *C. elegans* and 21,936 in *C. briggsae* (Table 2). The genome-wide unspliced transcript footprint in *C. remanei*, comprising the combination of exons, introns and untranslated regions (UTR’s), comprised ~69.33Mb (58.51% of the assembled genome; 52.92% of the estimated genome size), which is 18.83% larger than the equivalent footprint in *C. briggsae* (58.09Mb [28], 53.79% of the assembled genome), 18.74% larger than *C. elegans* (58.39Mb [20], 58.16% of the assembled genome). Consequently, the transcribed genic footprint explains the difference in assembled genome size between *C. briggsae* and *C. remanei* and ~60% of the difference in assembled genome size between *C. elegans* and *C. remanei*. Similarly, the outcrossing *C. brenneri* has 30,667 protein-coding genes and an unspliced transcript footprint of 70.5Mb (Fig 1), although these are likely overestimates due to allelism in the assembly [19]. Consistent with this pattern, we predict 22,326 coding genes within the self-fertile *C. tropicalis* (see Table 1 for all species). These results are consistent with an analysis of gene content within these species assessed by whole-genome transcriptional analysis [31]. Thus, while there is no evidence for differences in repeat content or indel biases among these species, there is strong evidence that genome size differences result from differential protein-coding gene content in self-fertile and outcrossing *Caenorhabditis*.

**Table 2:** Summary of gene-specific properties across species.

		*C. elegans*	*C. briggsae*	*C. remanei*
Autosomes (bp)	Mean Exon	1,213	1,362	1,335
	Mean Intron	1,221	1,610	2,393
	Mean Total	2,649	3,120	3,831
	Intergenic	1,915	2,192	2,598
	Median Exon	999	1,047	1,335
	Median Intron	740	839	1,186
	Median Total	1,896	2,025	2,277
	Intergenic	812	1,062	1,059
Gene Size– X Chr (bp)	Mean Exon	1,254	1,409	1,322
	Mean Intron	1,450	1,586	1,419
	Mean Total	2,924	3,145	2,856
	Intergenic	3,185	3,153	2,356
	Median Exon	984	1,095	966
	Median Intron	1,208	1,066	851
	Median Total	2,351	2,296	1,922
	Intergenic	1,671	1,880	1,444

### Intergenic distances

**Fig 3:**
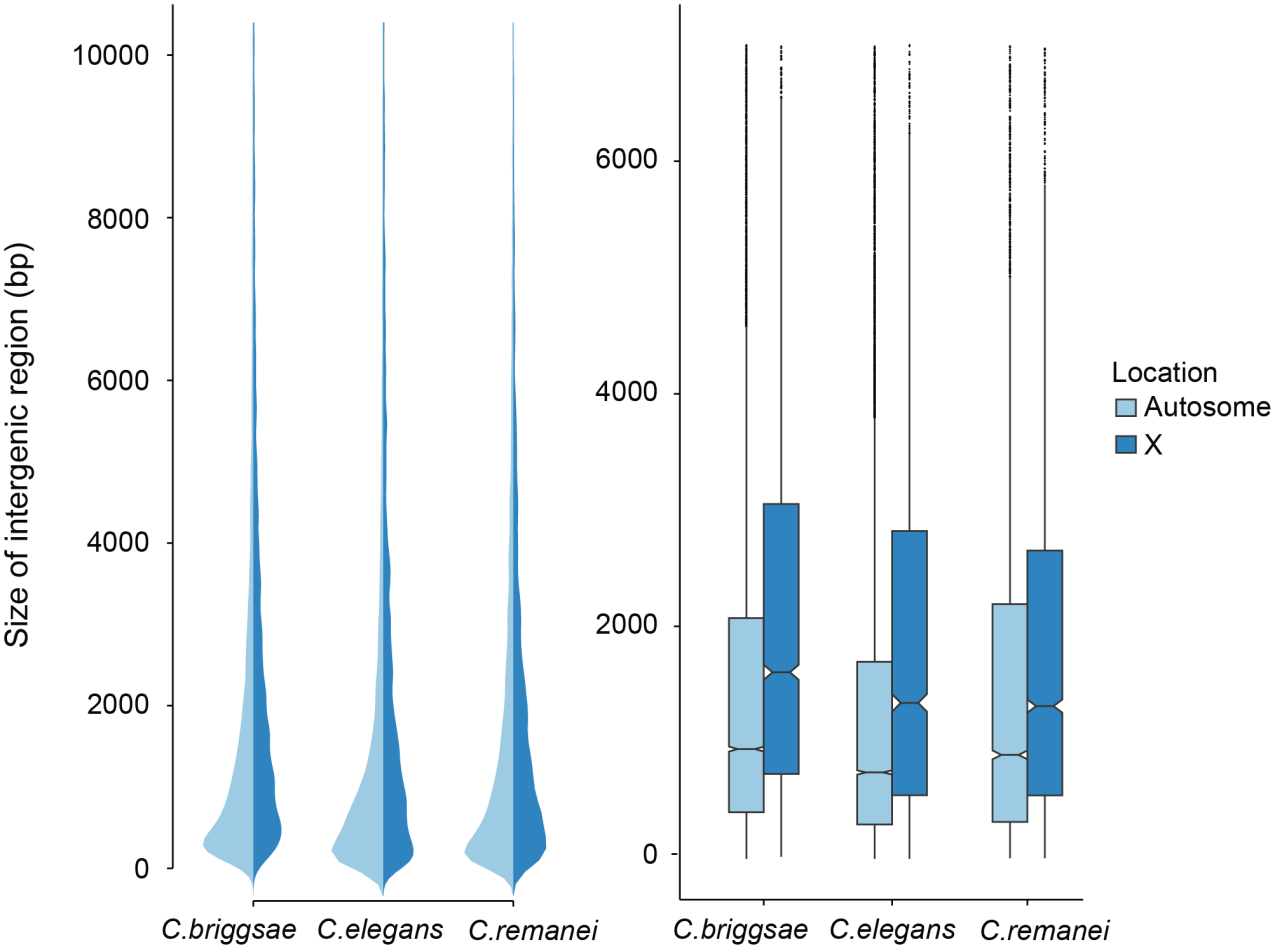
Comparison of intergenic spaces between autosomes and X chromosomes. (a) Kernel smoothed distribution of intergenic spaces across the entire genome for *C. elegans*, *C. briggsae* and *C. remanei*. (b) Intergenic spaces differ between autosomes and the X chromosome in *C. briggsae* (Kruskal-Wallis *χ* ^2^ = 556.09, *df* = 1, *p* < 2*x*10^−16^), *C. elegans* (Kruskal-Wallis *χ* ^2^ = 476.32, *df* = 1, *p* < 2*x*10^−16^) and *C. remanei* (Kruskal-Wallis *χ* ^2^ = 76.76, *df* = 1, *p* < 2*x*10^−16^). The boxplot indicates the bottom and top quartiles (black lines), middle quartiles (blue boxes), and median value (central notch) with outliers are shown as black dots. Intergenic spaces differ significantly between species on autosomes (Kruskal-Wallis *χ* ^2^ = 328.4957; *df* = 2, *p* < 2*x*10^−16^; Bonferroni-adjusted Pairwise Wilcoxon Rank Sum *C. remanei*:*C. elegans* *p* < 2*x*10^−16^, *C. remanei*:*C. briggsae* *p* < 0.039; *C. briggsae*:*C. elegans* *p* < 2*x*10^−16^) and the X chromosome (Kruskal-Wallis *χ* ^2^ = 112.52, *df* = 2, *p* < 2*x*10^−16^; Bonferroni-adjusted Pairwise Wilcoxon Rank Sum *C. remanei*:*C. elegans* *p* < 1.6*x*10^−7^, *C. remanei*:*C. briggsae* *p* < 2*x*10^−16^; *C. briggsae*:*C. elegans* *p* < 0.0005).

Intergenic distances vary widely within *Caenorhabditis* genomes, with some genes located in co-transcribed operons that are separated by a few nucleotides and other genes separated by many kilobases of sequence. Autosomal intergenic spacing for *C. remanei* exceeded that of both *C. briggsae* and *C. elegans*, despite these being lower bound values for *C. remanei* because of the potential for unincluded, unassembled regions probably underestimate *C. remanei* intergenic distances (Fig 3; Table 2). Across the entire genome (including scaffolds not included in linkage groups) the total intergenic content of *C. remanei* was 0.79Mb larger than that of *C. briggsae* and 10.73Mb larger than that of *C. elegans*.

In an outcrossing population with a 50:50 sex ratio there are 3/4 the number of X chromosomes in a population for any given autosome. The effective population size of the X is also reduced by variance in male mating success, and under the effective population hypothesis these forces should result in increased genetic drift and the proliferation of weakly deleterious elements [6]. In contrast, the effective population size of the X chromosome should be equivalent to the autosomes in self-fertile organisms [41]. However, we found no difference in the relative ratio of intergenic regions in the selfing versus outcrossing species (Fig 3). Presumably either the evolutionary forces that are responsible for maintaining variation in the size of intergenic spaces are not sensitive to changes in effective population size, the timescale since the advent of selfing has not been long enough for the genomic features of the sex chromosome and autosomes to equilibrate in the selfers [23], or, as we discuss below, there are other genetic differences between these chromosomes that drive this pattern.

### Divergence in gene structure

**Fig 4:**
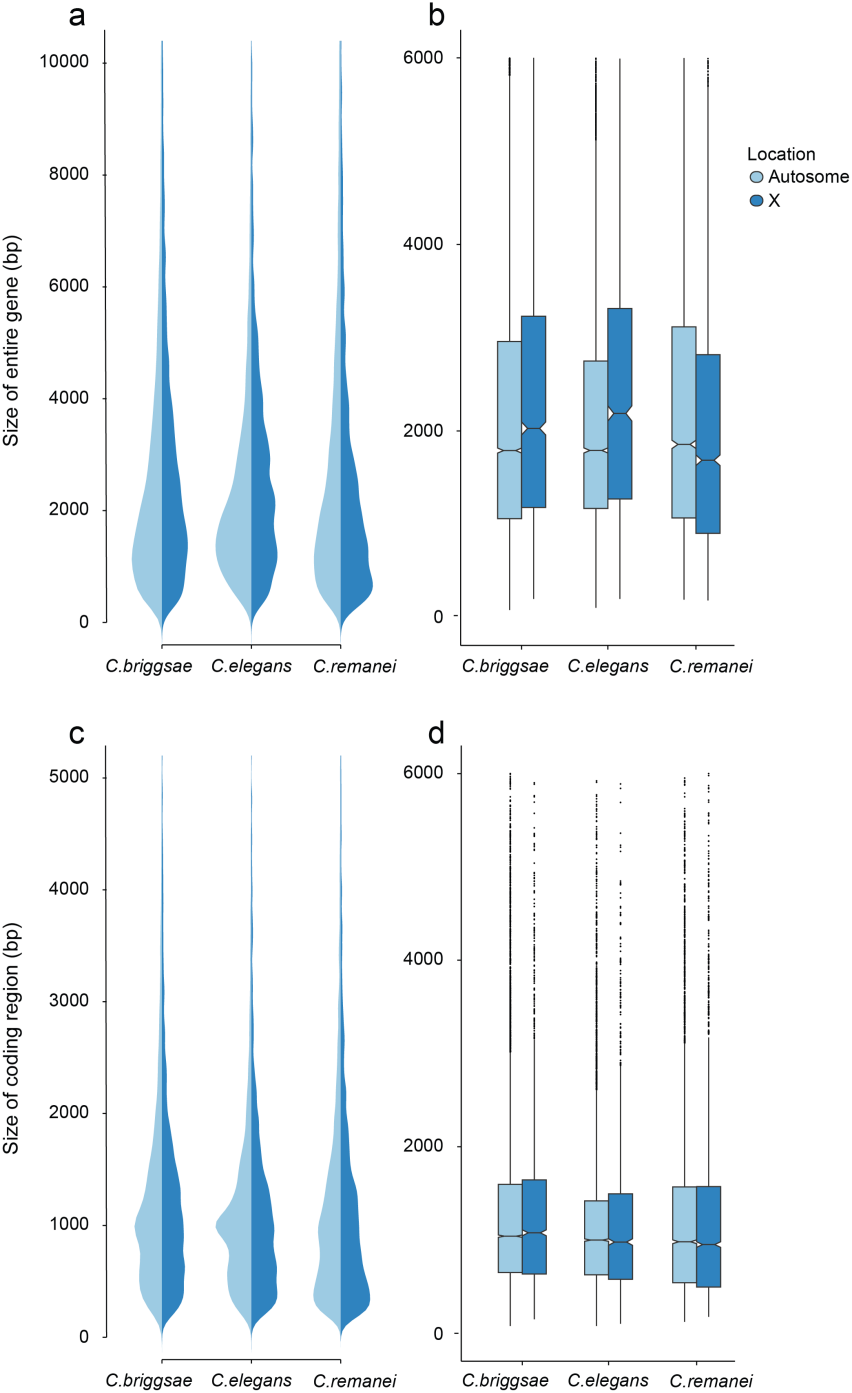
Differences in total gene size (introns and exons) versus protein coding size (exons) in *C. elegans*, *C. briggsae* and *C. remanei*. (a-b) Gene Size differs between autosomes and the X chromosome in *C. briggsae* (Kruskal-Wallis *χ* ^2^ = 24.63, *df* = 1, *p* < 6.96*x*10^−7^), *C. elegans* (Kruskal-Wallis *χ* ^2^ = 58.04, *df* = 1, *p* < 2.56*x*10^−14^) and *C. remanei* (Kruskal-Wallis *χ* ^2^ = 99.10, *df* = 1, *p* < 2*x*10^−16^) but protein size does not (*C. briggsae* Kruskal-Wallis *χ* ^2^ = 0.94, *df* = 1, *p* = 0.66; *C. elegans* Kruskal-Wallis *χ* ^2^ = 0.29, *df* = 1, *p* = 1; *C. remanei* Kruskal-Wallis *χ* ^2^ = 4.3096, *df* = 1, *p* = 0.08). Gene size differs significantly among the species on autosomes (Kruskal-Wallis *χ* ^2^ = 152.86; *df* = 2, *p* < 2*x*10^−16^; Bonferroni-adjusted Pairwise Wilcoxon Rank Sum *C. remanei*:*C. elegans* *p* < 2*x*10^−16^, *C. remanei*:*C. briggsae* *p* < 2*x*10^−16^; *C. briggsae*:*C. elegans* *p* < 6*x*10^−5^) and between *C. remanei* and the self-fertile hermaprodites on the X chromsome (Kruskal-Wallis *χ* ^2^ = 64.39; *df* = 2, *p* < 1*x*10^−14^; Bonferroni-adjusted Pairwise Wilcoxon Rank Sum *C. remanei*:*C. elegans* *p* < 2*x*10^−10^, *C. remanei*:*C. briggsae* *p* < 1.8*x*10^−11^; *C. briggsae*:*C.elegans* *p* = 1). (c-d) Protein size differs significantly between *C. briggsae* and *C. elegans* and *C. briggsae* and *C. remanei* on both the autosomes (Kruskal-Wallis *χ* ^2^ = 91.32; *df* = 2, *p* < 2*x*10^−16^; Bonferroni-adjusted Pairwise Wilcoxon Rank Sum *C. remanei*:*C. elegans* *p* = 1, *C. remanei*:*C. briggsae* *p* < 2*x*10^−16^; *C. briggsae*:*C.elegans* *p* < 1.5*x*10^−11^) and X chromosome (Kruskal-Wallis *χ* ^2^ = 40.36; *df* = 2, *p* < 1.7*x*10^−9^; Bonferroni-adjusted Pairwise Wilcoxon Rank Sum *C. remanei*:*C. elegans* *p* = 0.92, *C. remanei*:*C. briggsae* *p* < 4*x*10^−9^; *C. briggsae*:*C.elegans* *p* < 2*x*10^−5^).

In order to analyze gene structure, we identified a set of co-orthologs conserved as pairs between *C. remanei*, *C. briggsae* and *C. elegans* (Fig 4). We found that among our co-ortholog coding sequences the average number (6) and length (~200bp) of exons per gene was similar for the three species. The total length of gene transcripts was longer on autosomes in *C. remanei* than in *C. briggsae* or *C. elegans* but smaller on the X chromosome in *C. remanei* than in *C. briggsae* or *C. elegans* (Fig 4, Table 2). The protein sequences were not significantly different between the X chromosome and autosomes in any of the three species and among the species only *C. briggsae* had protein sizes that differed significantly from the other species.

Thus there is an interesting interaction between chromosomal gene structure and mating system, with intron size expanding on the X with the advent of selfing. Although this could reflect an accumulation of slightly deleterious DNA in the selfing species because of a decrease in effective population size [42], we would expect the same expansion to occur within autosomes. Another alternative is that increased selection on male function on males within *C. remanei* in turn drives stronger X-specific genomic evolution, although this seems unlikely given the fact that it appears that the X is actually enriched for genes affecting female/hermaphroditic function as opposed to male function [43].

Given that these explanations based purely on population genetics do not appear to fit the data, another explanation based on genetic differences between the chromosomes seems more likely. In *C. elegans*, it is known that genetic map is much more uniform on the X chromosome than on the autosomes (which tend to have very little recombination toward their centers)[44]. Indeed the molecular machinery that generates chromosome pairing and crossing over is different for the X than the autosomes [45]. Because the effective recombination rate is lower in selfers than in outcrossers, the difference in intron size between the X and autosomes within *C. elegans* and *C. briggsae* may reflect differential sensitivity to changes in effective recombination across chromosomes, coupled with the fact that the X and autosomes now experience similar effective population sizes under selfing. There may therefore be a complex interaction between recombination, drift and selection on the X that is driving this unusual pattern. Distinguishing among hypotheses will require a more careful analysis of the pattern of selection operating on the X and autosomes.

### Functional divergence

**Fig 5:**
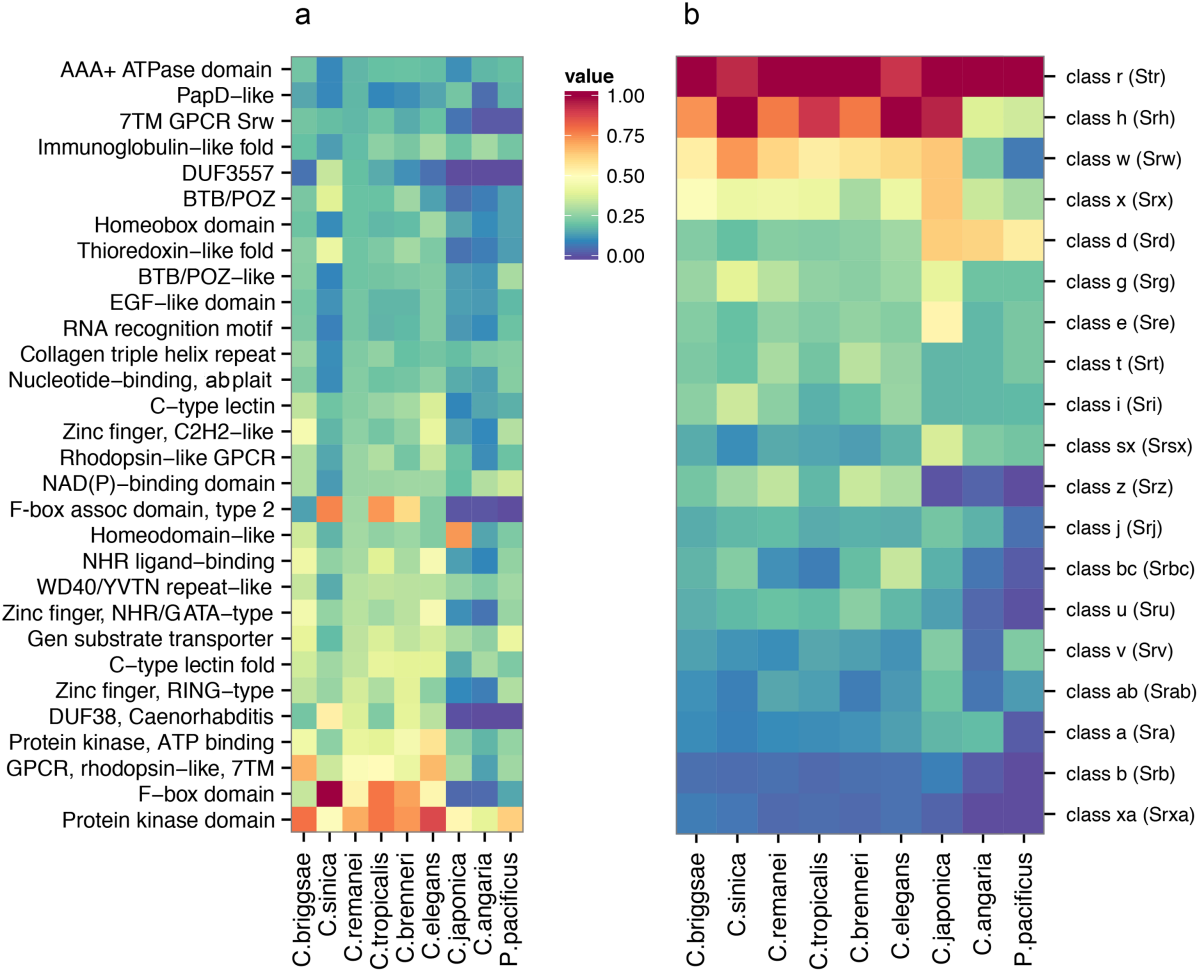
Comparative analysis of protein diversity. (a) The 30 most significantly enriched protein domains (*p* < 0.001) in the *C. remanei* genome (as compared to the *C. elegans* genome), and the corresponding interproscan annotations across *Caenorhabditis* and *P. pacificus*. The value shown is scaled relative to the top protein domain identified in each species. The species are plotted in phylogenetic order and protein kinase domains, F-box domains, GPCRs, domain of unknown function 38, and protein of unknown function DUF3557 are found in low numbers outside the *Elegans* group. (b) The relative representation of each 7TM GPCR family across *Caenorhabditis* and *P. pacificus*. The species are plotted in phylogenetic order and each value is scaled relative to the top 7TM GPCR family identified in each species.

*Caenorhabditis* genomes have large numbers of nematode-specific and species-specific proteins [46], and high divergence makes it difficult to conclusively identify individual genes that are present in outcrossing *Caenorhabditis* but lost in the selfers. To accommodate this, we characterized functional divergence between self-fertile and outcrossing *Caenorhabditis* by analyzing putative protein domains in the genomes of *Caenorhabditis* and the distantly related *P. pacificus* (Fig 5). We found no functional groups that were significantly enriched in the outcrossing *Caenorhabditis* relative to the selfing *Caenorhabditis*. There are numerous species-specific differences, however. For example, we identified between 191 and 1,721 proteins with F-box domains (IPR001810) in *C. briggsae*, *C. sinica*, *C. remanei*, *C. tropicalis*, *C. brenneri* and *C. elegans*, 5–18 times as many as identified in *C. japonica*, *C. angaria* and *P. pacificus*.

Domains found in large numbers in members of the Elegans supergroup encompass functionally diverse proteins (*i.e.*, protein kinase domains, C-type lectins, and zinc fingers), and proteins known to be important in *Caenorhabditis*, including the 7TM GPCRs introduced earlier, F-box domains, and the *Caenorhabditis*-specific Domain of Unknown Function DUF38 (Fig 5A). These proteins are clearly fundamental for *Caenorhabditis* biology, but for the most part there is little functional information known about these rapidly evolving protein families. To more closely examine a particularly hyper-diverse gene family, we identified 1,499 serpentine receptor genes in *C. elegans*, 1,125 in *C. briggsae* and 1,026 in *C. remanei* and the relative distribution among the families is similar across the Elegans supergroup (Fig 5B). Despite these large numbers there is functional evidence, direct or inferred, for only ~10 GPCRs [47]. Further analysis of similarities across whole molecular pathways are provided in the Supplementary Material (S1 Text, S3 Fig). We did find a small number of genes that are present in outcrossing species but lost in within selfing species, although no obvious classes of genes reveal themselves as being specifically lost within these groups (S1 Text). Overall, then, broad genomic comparisons do not reveal any systematic gain or loss of functional categories within and between mating system types, although each individual genome can show dramatic differences within any given gene family.

### Evolution of genome size under selfing

Within-species polymorphism across *Caenorhabditis* varies by several orders of magnitude, with selfing species being relatively depauperate of variation [16] and outcrossing species being among the most polymorphic animals yet observed [18]. At least one likely reason for these dramatic differences in polymorphism are differences in effective population size among species, which has been estimated as <10,000 in *C. elegans*[48], <60,000 in *C. briggsae*[49] and >1,000,000 in *C. remanei*[17]. We would therefore expect that deleterious elements would be more likely to accumulate and expand the genomes of self-reproducing species because of their small population sizes. Instead, we find just the opposite. Genomes are smaller in selfers and intergenic spaces and protein-coding genes are larger on the X chromosome than autosomes. In the outcrossing *C. remanei* protein-coding genes are smaller on the X chromosome despite the reduced effective population size of the X versus the autosomes in male-female species.

The transition from outcrossing to self-fertilization is common in plants, and plant genome size is similarly positively correlated with outcrossing [9], although this relationship is weakened when corrected for phylogenetic relatedness [50]. It is possible that changes in genome size may be a consequence of ecological shifts that accompany life history differences being selfing and outcrossing species. However, the genome of the model self-fertile *Arabidopsis thaliana* (125Mb) is smaller than its outcrossing relative *A. lyrata* (207Mb) largely due to numerous small and large-scale deletions, including 17% fewer genes [30]. TEs play a major role in plant genome size evolution [24, 51, 52] and, while the genome of *A. lyrata* does show an increase in TE activity relative to *A. thaliana*, a comparison between *Capsella rubella*, a plant that became self-fertile less than 200,000 years ago, and the related outcrossing *C. grandiflora* reported few differences in TE content [53]. We find no evidence that the genome size differences between selfing and outcrossing species are mediated by TE activity and/or other forms of small indels. Instead, DNA loss in *Caenorhabditis* appears to have occurred specifically at the level of individual genes.

Natural selection might drive genome reduction, or genome shrinkage could accrue through deletion biases and genetic drift. DNA content is positively correlated with increased cell size and negatively correlated with cell growth and division, metabolic rate [54], and developmental rate [32], but it is unclear why self-fertility would necessarily lead to increased selection on these traits. Alternatively, selective sweeps on new beneficial mutations could lead to the fixation of weakly deleterious deletions because of increased linkage disequilibria in selfers. The fixation of such deletions would be particularly facilitated in a non-adaptive manner if deletion per se acted as a directional process. For example, deletions predominate over insertions in *C. briggsae* nuclear [55, 56] and mitochondrial DNA [57], however insertions predominate over deletions in *C. elegans* mutation accumulation lines [55, 58, 59] and under temperature stress [56]. Perhaps most interestingly, there is evidence in *C. elegans* that autosomal deletions are preferentially transmitted to X-bearing sperm (and thereby hermaphrodites in this XO sex determination system)[37]. This kind of bias could rapidly reduce genome size following the transition to self-fertilization. However, rather than observing systematic reduction in gene size with the selfing species, we find that both *C. elegans* and *C. briggsae* have larger introns on the X than *C. remanei* while maintaining similarly sized genes on the autosomes. Thus, while there are definitely deletions across the whole genome, they are at the level of whole genes instead of being randomly spread across functional elements such as introns, as would be expected if the genome size reduction were driven by a directional mutation process.

Instead, it appears that adaptation to self-fertility per se is the most likely explanation for the reduction in genome size. For instance it may be advantageous to lose/alter systems directly related to maintaining outcrossing, such as mating in the case of nematodes or floral characteristics in the case of plants [23, 60]. In keeping with this, Thomas et al.[31] found that genes with higher degrees of sex-specific expression tend to be lost more frequently than other genes within these same species. Similar loss of function changes appear to be common in other cases of adaptive phenotypic evolution [61]. Overall, then, these results suggest an evolutionary model for genome reduction following the evolution of selfing within this group of nematodes: 1) relaxed selection on specific genes, like those involved in facilitation of outcrossing [31, 53]; 2) deletion of genes and their surrounding intergenic sequences; and 3) accumulation of these deletions resulting in derived decreases in genome size. There are several other genera of nematodes within this family that also show variation in mating systems [62, 63], so it should be possible to discern if this is a general pattern of genome loss within self-fertilizing species.

Lynch [42] proposed the effective population size hypothesis as a first step in transforming “the descriptive field of comparative genomics into a more mechanistic theory of evolutionary genomics.” Our results indicate that self-fertile organisms experience loss of DNA as a general feature of the transition in mating systems but that these losses are not driven by changes in effective population size per se. The specific mechanisms by which this occurs appear to vary across groups, from plants to animals. Linking changes within specific genomic functional classes with the dynamics of natural selection and fitness differences that favor DNA loss in self-fertile organisms is the next step in understanding the influence of mating system on genome evolution.

## Materials and Methods

### Worm culture and strain construction

*C. remanei* strains were cultured using standard methods adopted from techniques developed for *C. elegans*[64]. The *C. remanei* PX356 strain was created from the canonical isofemale line EM464 originally isolated from New York (USA), which was initially named *C. vulgaris* but has since been synonymized with *C. remanei*[65]. The existing polymorphic assembly of *C. remanei* is based on a partially inbred line derived from this same strain (EM4641). To overcome the extreme inbreeding depression usually observed within *C. remanei*, we derived 200 independent lines from the original EM464 population and subjected them to brother-sister mating in order to allow the independent fixation and loss of as many recessive deleterious alleles as possible. All but two of the lines went extinct by generation 7. These two remaining lines were then crossed together and maintained as an outcrossing population for 20 generations to increase the probability that deleterious alleles alternatively fixed in the two lines could recombine. An additional 100 lines were derived from this secondary population and then subject to brother-sister mating for 23 generations. The sole surviving line from this procedure was deemed the new canonical EM464-derived line: PX356. In order to create an alternative mapping line, a similar procedure was conducted for an isofemale line (PB259) originally collected from a forest in Ohio (USA) by Scott Baird (Wright State University). This resulted in an additional, divergent inbred line: PX439.

### Isolation of DNA and RNA

Genomic DNA was isolated from either starved L1 larvae following a bleach “hatch-off” or from mixed stage populations from a sucrose float using the DNeasy Blood and Tissue kit (Qiagen) with the *C.elegans* supplemental protocol. Total RNA was isolated from mixed stage populations using Trizol (Invitrogen). mRNA was purified using Dynabeads Oligo d(T)25 (Invitrogen) and fragmented (Ambion) before cDNA synthesis [66].

### Next-generation sequencing

Paired-end genomic DNA sequencing libraries were constructed using the Nextera DNA sample preparation kit (Illumina) or the NEBNext DNA library kit for Illumina (NEB) as per the manufacturers protocols. Using the NEBNext kit, genomic DNA was fragmented with a Bioruptor sonicator (Diagenode) set on high for ten 30 second ON/OFF cycles. Final libraries were size selected on 2% agarose gels with an average genomic insert size of 180 bp as per ALLPATHS-LG recommendations. All libraries were quantified by qPCR (Life Technologies) and the proper size range was confirmed using a fragment analyzer (Advanced Analytical Technologies). Libraries were sequenced as 2 X 101nt reads using an Illumina HiSeq instrument.

The mate pair libraries were constructed using standard molecular techniques following the manufacturers recommendations. In brief, genomic DNA was sheared on the low setting for 5 seconds using a Bioruptor sonicator (Diagenode) and purified using the Desalting and Concentrating DNA section for the QIAEX-II kit (Qiagen). DNA was end-repaired using the End-it kit from epicentre. Following purification, the DNA was biotin labelled with 1mM dNTP (4% biotin), purified and run on a 0.6% agarose gel. Size ranges of 3, 5 and 7 kb were isolated and purified using the standard QIAEX II kit. Circularization was carried out overnight at 16 deg.C with ~200ng DNA using T3 ligase (Enzymatics) with T4 ligase buffer. The non-circularized DNA was digested with 3*μl* of DNA exonuclease (NEB) and placed at 37 deg.C for 20 min. and heat inactivated for 30 min. at 70 deg.C. The DNA was then sheared to ~400 bp using a Bioruptor for ten 30 sec. ON/OFF cycles at high and then purified. The biotin labelled DNA pieces were isolated using Dynabeads M-280 strepavidin (Invitrogen). While on the beads, the DNA fragments were end-repaired, A-tailed, and t-overhang adapters were added before PCR enrichment for 15 cycles using Phusion polymerase (NEB). Libraries were isolated from 2% agarose gels at ~ 400 bp average size range and eluted in EB buffer with 1% tween 20. Final libraries were validated for correct size and molar concentration as noted above. Mate-paired libraries were sequenced as 2 X 101nt reads using an Illumina HiSeq instrument (cDNA synthesis and RNA sequencing libraries were prepared as previously reported [66]).

### Genome assembly

We constructed the *C. remanei* assembly from ∼ 560x coverage of 180bp paired end fragments designed to have overlapping reads on both ends. This high depth of coverage will necessarily include a large number of sequencing errors which would have complicated assembly, and a large number of repeat elements which would have increased assembly time. We pre-filtered the 180bp paired end fragments by kmer frequency spectra (here, k = 15) to address these biases. We removed reads with greater than 12 rare kmers (singletons) to eliminate possible errors and reads with greater than 51 abundant kmers (occurring more than 20,000 times in our dataset) to eliminate possible over-represented repeats (additional details are given in S1 Text). The final short-read dataset averaged 416x coverage across the estimated genome size (131Mb). We also used mate pair libraries with inserts of varying sizes: 31.5x coverage of 0.7–2kb insert paired end fragments, 29x coverage of 2–4kb insert paired end fragments, and 15x coverage of 4–7kb insert paired end fragments. We sequenced 101bp reads with 6bp inline barcodes which resulted in 95bp sequences.

We used the assembly software ALLPATHS-LG [67], which performs its own kmer spectra correction of sequencing reads (k = 25), uses a de Bruijn graph algorithm to build contiguous sequences from the 180bp reads, and constructs scaffolds with mate pair sequences. The initial heterozygosity rate was estimated as 1/176bp and we used the haploidify option to address residual heterozygosity in our inbred strain (S3 Table). We used a multi-step decision tree to identify possible contaminant sequences and removed 17Mb of contaminant scaffolds (details are given in S1 Text; S4 Fig). After assembly we aligned the paired end sequences to our final linkage groups and scaffolds with GSNAP [68] and used SAMtools [69] to summarize single nucleotide polymorphisms (SNPs) in a.vcf (variant call format) file. We analyzed this file with a custom pipeline of perl scripts that divide the annotated genomic regions into introns, exons, transposable elements, intergenic regions, transcription start sites, 5’ UTRs and 3’ UTRs and measures the number of polymorphic sites in each of these categories. Read alignments were discarded if the per-base sequencing coverage exceeded 600 (as these may be poorly assembled, collapsed repetitive regions) or if the base quality fell below 90% certainty (Q10 Phred+33). Our estimated residual polymorphism therefore applies only to the well-assembled genic regions and excludes intergenic sequences, transposable elements and other poorly assembled repeats.

Our kmer frequency spectra filtering removed over-represented sequences. In order to eliminate the possibility that this filtering may have created a bias in assembling repetitive elements we also *de novo* assembled the entire set of 180bp paired end fragments and mate pair libraries with ALLPATHS-LG [67]. We analyzed the repeat content of this genome sequence (methods are given below in the section **Characterizing repeats**) and found that the repeat content of this sequence was lower than our kmer-filtered genome sequence (13.92% vs. 15.73%). The kmer-filtered assembly was 118.5Mb with 4Mb of gapped sequence and 1,600 scaffolds (S3 Table). The unfiltered assembly was similar in size (119.1Mb) but contained 7Mb of gapped sequence and was fragmented into 3,921 scaffolds. We concluded that kmer frequency spectra did not specifically eliminate repeat elements from our genome but it did remove noise and facilitate a higher-quality, ungapped genome sequence.

### Genetic map construction

We generated 64 recombinant inbred lines (RILs) from a cross between parental strains PX356 and PX439 following an advanced intercross method [70]. We then used restriction site associated DNA (RAD) markers [71], generated with EcoRI, to identify SNP markers within each of these strains, as well as in the parental lines. We used the software *Stacks*[72] to assign sequencing reads to genetic loci and identified 25,447 mappable (*i.e.*, *AA x BB* where *A* is the PX356 genotype and *B* is the PX439 genotype) polymorphic markers. The parental strains appear to be partially reproductively isolated and so many of these SNP markers showed extensive segregation distortion (S5 Fig). We used SAMtools [69] to align the markers to our assembled scaffolds and identified scaffolds where >5 markers had parental genotype frequencies of >80% or <20% in the RILs. We eliminated all markers located on these scaffolds, markers that contained only duplicate data and those with low representation in the RILs (present in <40 lines). We constructed a genetic map in R/qtl [73] from the resulting 330 distinct SNP markers (S6–S7 Figs). We added markers with duplicate data back to the dataset and the final genetic map contains 2,688 SNP markers across 65 Mb (S8–11 Figs). The genetic map identified 4 scaffolds that were incorrectly joined in the assembly, and we broke these on the basis of parental genotype frequency in the RILs and synteny with the existing *C. remanei* draft assembly. We aligned the 3 large linkage groups to the *C. elegans* and *C. briggsae* chromosomes X, II, and IV but smaller linkage groups were not definitively assigned to chromosomes.

### Characterizing repeats

The PX356 genome was repeat masked with RepeatMasker v4.0.5 [74] using a custom *C. remanei* repeat library created by RepeatModeler v1.0.8. In order to compare repeat content between nematode species we also created custom repeat libraries for *C. briggsae*, *C. sinica*, *C. tropicalis*, *C. brenneri*, *C. elegans*, *C. japonica*, *C. angaria*, and *P. pacificus*, and repeat masked each genome with the custom repeat library and RepeatMasker. We compared these custom repeat characterizations to the repeats currently annotated in each genome (Wormbase release WS244) and found that each custom characterization was within 1–2% of the currently annotated content with the exception of *C. japonica* for which we predicted 42% repeat content. This is consistent with previous findings that the allelism in the *C. japonica* assembly produced over-representation of repeat regions [19]. We present the currently annotated content in Fig 1.

### Genome annotation and orthologous gene identification

We sequenced mRNA from our PX356 nematodes with a Illumina Hi-Seq machine and generated 16,250,052 paired end sequences. We assembled these data with the genome-independent Trinity RNA-seq assembly software [75] to generate a set of putative transcripts. We used the software MAKER2 [76] to annotate putative protein-coding loci, with the Trinity transcript set as EST evidence and the protein sequences from *C. briggsae*, *C. elegans*, *C. brenneri*, and the Uniprot/Swiss-Prot [77, 78] database (with *Caenorhabditis* removed) as protein homology evidence. The maximum intron size was specified as 5,000 nucleotides for evidence alignments. We used SNAP [79] and Augustus [80]*ab initio* gene prediction software within MAKER2 to generate a putative set of predictions, and used these initial predictions to re-train SNAP and Augustus to produce *C. remanei*-specific gene prediction models. We predicted 26,339 transcripts from 25,415 protein-coding genes and used a manually curated set of 169 miRNA sequences to annotate putative miRNA loci. Roughly half of the annotated genes (12,323) reside on the 13 linkage groups. Although the assembly is 9.5% shorter than the flow-cytometry estimated genome size, analysis of a core set of genes that are thought to be conserved in single copy in eukaryotes [81] indicates that 95.16% are in complete form in the assembled sequence and the remaining 4.84% are partially complete.

We used the same annotation pipeline to annotate protein-coding genes in the genomes of *C. angaria*, *C. brenneri*, *C. briggsae*, *C. elegans*, *C. japonica*, *C. sinica* and *C. tropical*. We found that the re-annotation estimated a similar protein-coding footprint with the exception of *C. brenneri*, for which we predicted >15Mb additional genic content. This is consistent with previous findings that >30% of the genes in the *C. brenneri* genome are found in two copies [19].

We identified protein motifs and domains with InterProScan [82] (version 5.3), which searches against public protein databases including ProDom [83], PRINTS [84, 85], Pfam [86], SMART [87], PANTHER [88] and PROSITE [89]. We used the Repbase (version 16.10) database of repetitive elements and a library of *de novo* repeats identified with RepeatModeler [29] to analyze transposable elements and genomic repeat content. We used OrthoMCL (version 1.4) to identify orthologous gene clusters between *C. remanei*, *C. briggsae*, *C. elegans*, and *C. brenneri*. The *C. brenneri* genome assembly is highly fragmented (3305 scaffolds) and conflated by ~30% allelic assembly artifacts to be 55Mb larger than the flow-cytometry estimated genome size [19], but is the only other outcrossing *Caenorhabditis* in the Elegans group with a genome sequence suitable for comparative analyses. Briefly, OrthoMCL [90] uses BLASTP [91] to calculate pairwise protein sequence similarities, and Markov clustering of the similarity scores to define orthologous proteins among the species and paralogous proteins within each proteome.

### Characterizing genome content

In order to compare genome content between nematode species we used our MAKER2 gene annotation pipeline to identify protein-coding genes in the Wormbase release WS244 genome sequences of *C. briggsae*, *C. sinica*, *C. tropicalis*, *C. brenneri*, *C. elegans*, *C. japonica*, *C. angaria*, and *P. pacificus* (S2 Table). We found that each custom characterization was within 3–5% of the currently annotated content, with the exception of *C. brannier* for which we predicted a larger gene content. This is consistent with previous findings that >30% of *C. elegans* orthologous genes are found in two copies in the *C. brenneri* assembly [19]. We present the currently annotated content in Fig 1.

### Measuring intergenic spaces, gene size, and protein size

We used BedTools v2.22.1 [92] to identify intergenic spaces, genic regions, and exonic regions in the genome sequences of *C. elegans*, *C. elegans*, and *C. remanei*. We used the statistical computing language R to calculate descriptive statistics, Kruskal-Wallis rank sum tests, and Pairwise Wilcoxon rank sum tests. We were not able to use parametric statistics because our data were nucleotide counts, each of the distributions showed severe heteroscedasticity, and the sample sizes were unbalanced. Nonparametric statistical tests can not address interaction terms or multi-factor comparisons and we performed one-way tests and Bonferroni corrected our p-values for multiple comparisons.

### Accession codes

Assembly and annotation are at www.wormbase.org and are deposited in GenBank under BioProject ID PRJNA248909. De novo annotations of existing genomes are published at figshare.com at dx.doi.org/10.6084/m9.figshare.1399184, dx.doi.org/10.6084/m9.figshare.1396472, dx.doi.org/10.6084/m9.figshare.1396473, dx.doi.org/10.6084/m9.figshare.1396474, dx.doi.org/10.6084/m9.figshare.1396475, dx.doi.org/10.6084/m9.figshare.1396476, dx.doi.org/10.6084/m9.figshare.1396477, and dx.doi.org/10.6084/m9.figshare.1396478. Our analysis pipeline is available at github.com/Cutterlab/popgenome_pipeline.

## Supporting Information

S1 Text Supplementary methods and analysis. Includes more detailed descriptions about the assembly and genetic mapping of the *C. remanei* genome, as well as details about analysis of repetitive elements and molecular pathways.
(PDF)

S1 Fig Analysis of the Wormbase *C. remanei* genome sequence. (PDF)

S2 Fig Aligned blocks of sequence between (A) *C. remanei* and *C. briggsae*, (B) *C. remanei* and *C. elegans*, and (C) *C. briggsae* and *C. elegans* do not show size bias. (PDF)

S3 Fig The 50 most significantly over- and under-enriched pathway components in the *C. remanei* genome as compared to the *C. elegans* genome. Each value is scaled relative to the top pathway identified in each species, and pathways are grouped by biological function. *C. remanei* has a significant overrepresentation of pathway components involved in cellular processes, nucleotide metabolism, lipid metabolism and amino acid metabolism and a significant underrepresentation of pathway components involved in neural development and carbohydrate metabolism.
(PDF)

S4 Fig Individual scaffolds were assigned *Caenorhabditis* or non-*Caenorhabditis* origin based on GC content and average per-base sequencing coverage. A) The training data used for the decision tree. B) Initial assignments identified scaffolds of *Caenorhabditis* or non-*Caenorhabditis* origin. C) 1589 scaffolds were identified as either *Caenorhabditis* or non-*Caenorhabditis* with high probability and 11 scaffolds had ambiguous origins (pictured in green above). For the final set of *C. remanei* DNA we included all scaffolds with p>0.2. There are three scaffolds that BLAST identified as of *Caenorhabditis* origin but the GC/coverage profile indicated non-*Caenorhabditis* origin. We included these in the final assembly as well for the sake of completeness.
(PDF)

S5 Fig Allele frequencies for every RAD-associated SNP marker. Markers are plotted along each scaffold and scaffolds are ordered by length.
(PDF)

S6 Fig Pairwise recombination fractions and LOD scores for the final genetic map. (PDF)

S7 Fig *C. remanei* genetic map. (PDF)

S8 Fig Relationship between physical and genetic maps for linkage groups 1–4. (PDF)

S9 Fig Relationship between physical and genetic maps for linkage groups 5–8. (PDF)

S10 Fig Relationship between physical and genetic maps for linkage groups 9–12. (PDF)

S11 Fig Relationship between physical and genetic maps for linkage group 13. (PDF)

S12 Fig The cumulative length distribution of the linkage groups and scaffolds. Roughly 50% of the 118.5Mb assembly is contained in 10 large linkage groups and scaffolds, and 90% of the length is contained in 160 linkage groups and scaffolds.
(PDF)

S1 Table Repetitive content and length aligned to the existing Wormbase *C. remanei* genome. (PDF)

S2 Table Genome content estimated from *de novo* annotation with our uniform pipeline. (PDF)

S3 Table Assembly size, Contig length, Scaffold length, N50 for Contigs and N50 for Scaffolds. (PDF)

S4 Table Interproscan protein annotation categories for proteins found in outcrossing *Caenorhabditis* (Elegans supergroup: *C. sinica*, *C. brenneri*, and *C. remanei*) but absent in the self-fertile *Caenorhabditis*. (PDF)
