# Correction: Reproductive Mode and the Evolution of Genome Size and Structure in *Caenorhabditis* Nematodes

**DOI:** 10.1371/journal.pgen.1005497

**Published:** 2015-09-22

**Authors:** Janna L. Fierst, John H. Willis, Cristel G. Thomas, Wei Wang, Rose M. Reynolds, Timothy E. Ahearne, Asher D. Cutter, Patrick C. Phillips

One of the URLs in the Data Availability statement is incorrect. http://dx.doi.org/10.6084/m9.figshare.1396475 is incorrect. The correct URL is http://dx.doi.org/10.6084/m9.figshare.1449264.

Additionally one of the species names is spelled incorrectly in the Materials and Methods section under the heading “Characterizing genome content”. It states *C*. *brannier*, whereas it should say *C*. *brenneri*. The corrected text is below.

In order to compare genome content between nematode species we used our MAKER2 gene annotation pipeline to identify protein-coding genes in the Wormbase release WS244 genome sequences of *C*. *briggsae*, *C*. *sinica*, *C*. *tropicalis*, *C*. *brenneri*, *C*. *elegans*, *C*. *japonica*, *C*. *angaria*, and *P*. *pacificus* (S2 Table). We found that each custom characterization was within 3–5% of the currently annotated content, with the exception of *C*. *brenneri* for which we predicted a larger gene content. This is consistent with previous findings that >30% of *C*. *elegans* orthologous genes are found in two copies in the *C*. *brenneri* assembly [19]. We present the currently annotated content in Fig 1.
