# Supplementary material for: Reproductive Mode and the Evolution of Genome Size and Structure in Caenorhabditis Nematodes
Source: PLoS Genet. 2015 Jun 26;11(6):e1005323. doi: 10.1371/journal.pgen.1005323 (PMC4482642; doi:10.1371/journal.pgen.1005323)
Supplement: S1 Text — Includes more detailed descriptions about the assembly and genetic mapping of the C. remanei genome, as well as details about analysis of repetitive elements and molecular pathways. (PDF) [file pgen.1005323.s001.pdf]

Supplementary Text

De novo sequencing and assembly of the *C. remanei* genome

We sequenced the PX356 genome at 560x coverage (735,363,194 sequencing reads). This high depth of coverage likely resulted in large numbers of sequencing errors, and increased computational time for assembly. To address these issues we used the *kmerfilter* program (part of the *Stacks* distribution [72]) to remove erroneous reads from our 180bp fragments. The program works by calculating each of the overlapping sequences of length k (k-mers) for every sequencing read. The frequency of individual k-mers can be used to identify problems in sequencing reads. Rare k-mers are likely sequencing errors while highly abundant k-mers are likely repetitive elements that increase computational time and can not be reliably assembled. For the data presented here, we tested a range of k-mer sizes from 9 to 17 and found that k=15 produced the highest N50 assembly with the greatest proportion aligning to the existing fragmented, duplicated *C. remanei* assembly available on Wormbase (S1 Table). We discarded all sequencing reads with greater than 12 rare k-mers and all reads with greater than 51 abundant k-mers. Rare is defined as occurring singly in the dataset and abundant is occurring greater than 20,000 times. We also discarded the sequencing pair for each discarded read, regardless of quality. The final 180bp fragment dataset was 416x coverage across the estimated genome 131Mb genome (589,349,020 sequencing reads).

We also experimented with assembling a lower number of 180bp fragments (1/2 the data), the ALLPATHS-LG [67] option "haploidify" which is meant to resolve residual polymorphism in diploid assemblies and k-mer filtering the mate pair data (S3 Table). We chose to assemble the entire dataset with the haploidify option and to not k-mer filter the mate pair sequences (28,802,880 sequencing reads) based on the N50 and proportion aligning criteria. The length we are able to align to the Wormbase *C. remanei* assembly is greater than what we were able to reliably confirm as *Caenorhabditis* DNA; we may be inaccurately discarding *Caenorhabditis* DNA as contaminants or there may be residual contamination in the Wormbase sequences.

Removing contamination and misassemblies

Our initial assembly was contaminated with GC-rich scaffolds of unknown origin. In order to identify possible contaminant sequences and/or misassembles in our ALLPATHS-LG [67] output, we used a multi-step decision tree implemented in Matlab (S4 Figure). First, we identified a set of 200 scaffolds that we could reliably assign to *Caenorhabditis* or non-*Caenorhabditis* (i.e., possible bacterial contaminant) origin based on BLAST [91] against the NCBI nucleotide collection (NR/NT). We used the initial 200 scaffolds as a training set and constructed a decision tree based on the GC content of the scaffold and the average per-base sequencing coverage. The decision tree then assigned a putative origin, *Caenorhabditis* or non-*Caenorhabditis*, to each scaffold and a

likelihood score with an associated p-value. Most of the scaffolds were easily identified as *Caenorhabditis* or non-*Caenorhabditis* based on these descriptors and we chose to include the ambiguous cases in the assembly for completeness. The final set contained 118.5Mb of sequence in 2,115 scaffolds (S12 Fig).

Genetic map construction

We used the *Stacks* [72] program to genotype the RAD sequences from our genetic mapping cross. *Stacks* analysis proceeds through a set of pipeline components: 1) sequenced reads from each individual are "Stacked" to form putative loci (here, we used the ustacks component for *de novo* locus discovery); 2) putative loci are loaded into a catalog using cstacks; and 3) these are matched against other individuals with sstacks. We used the cleaning script process radtags to correct single errors in the barcode or RAD site, and discarded sequences with two or more errors in either the barcode or RAD site. We ran the *Stacks* pipeline with the following parameters: 1) we required 3 reads to match exactly to form a locus in an individual; 2) we allowed a maximum distance of 3 SNP's for reads within a locus before splitting into two loci; 3) we permitted parental and progeny mismatching when constructing the catalog; and 4) we removed highly repetitive RAD-tags.

This analysis resulted in 218,252 RAD-tagged sequences across the 2 parental lines and 64 progeny. We removed loci if they did not contain a polymorphism, were found in less than 40 progeny, or were heterozygous or unmapped in the parental lines. This resulted in 25,748 SNP markers. These RAD-associated SNP markers showed marked segregation distortion in allele frequencies (S5 Figure). We used R/qtl [73] to construct an initial genetic map, but segregation distortion and linkage disequilibrium hampered map construction. We then culled the SNP markers for the least-distorted possible set of data (details are given in the main methods).

After culling our dataset we had 2,688 markers. We constructed initial linkage groups with a maximum recombination frequency of 0.0 and a minimum LOD of 12 to eliminate blocks of non-recombinant markers. This resulted in a set of 330 markers that aligned to 173 different scaffolds. We formed linkage groups from these 330 markers with a maximum recombination frequency of 0.35 and a minimum LOD of 6 and then calculated the number of obligate crossovers, the likelihood scores and the map size (in centimorgans) associated with alternating markers in a sliding window of 3 markers for the largest linkage group, 5 for the second and third largest, and 7 for the remainder of the linkage groups. We chose the marker order that minimized these three variables, and re-ordered markers to agree with the genomic scaffold coordinates (base pair location) when it did not affect these three variables (S6 Figure). We added sets of duplicate markers back to the final genetic map, and the final dataset (S7 Figure) contains 13 linkage groups and spans 65Mb of the assembly. Four small linkage groups contained only single scaffolds.

We plotted the physical location (bp along each scaffold) and genetic map location (cm) for each linkage group (S8-S11 Figures). The plots indicate some areas of disagreement between the scaffolds and genetic map, but we chose to leave these scaffolds as-is due to the low resolution of our map. A future *C. remanei* genetic map should be able to reliably distinguish between the physical and map locations. The final assembly contains 1,600 linkage groups and individual scaffolds (S12 Figure). Roughly 9% of the estimated *C. remanei* genome size is not covered in our assembly.

Gene annotation

We MAKER2 [76] version 2.31 to annotate putative protein-coding loci (details are given in the main text). Our final annotated genome contains 26,339 mRNA sequences derived from 25,415 protein-coding loci. We predict 148,740 exons, 8,458 5'-UTRs and 8,008 3'-UTRs. Due to the low coverage of our RNA-Seq dataset and extensive protein divergence between related *Caenorhabditis* [46] only 20,515 of our mRNA predictions are supported by either protein homology or our assembled transcripts. To validate the remainder of our gene models, we used GMAP-GSNAP [68] to align a dense set of single-end mRNA sequences generated from L1 *C. remanei* to our assembled genome and required that each sequence align to a maximum of one locus. The mRNA sequences from this experiment and the paired-end mRNA used in the annotations itself align to 25,546 mRNA sequences and 793 mRNA sequences are currently computational predictions without either mRNA-Seq support or protein homology. These may be inaccurate predictions, or they may be genes expressed at low levels or during other life stages. The number of unsupported genes is a small proportion of the total set (3.01%) and so we chose to retain these gene models in the final annotations.

Gene length distribution

We extracted two gene length datasets. The first contained all protein-coding genes predicted in our assembly, and the second contained all protein-coding genes predicted in our assembly with one or more defined orthologs (identified with OrthoMCL; details are given in the main methods) in *C. brenneri*, *C. briggsae* or *C. elegans*. We calculated the mean and median gene lengths for all protein-coding genes in both the first and second datasets. For the second dataset we aligned orthologous genes between *C. brenneri* and *C. remanei*, *C. briggsae* and *C. remanei* and *C. elegans* and *C. remanei* and calculated the ratio of the transcript length and the protein-coding sequence length. We calculated the intron length ratio as the remainder of these two quantities. We did not exclude genes with multiple co-orthologous counterparts in the other species, and these genes are multiply represented in the final calculations.

Intergenic space distribution

We extracted the coordinates of each protein-coding gene from the genome annotation files distributed for *C. briggsae* and *C. elegans*. We excluded genes that have not been placed on the genetic map, and calculated the intergenic space as the length between protein-coding genes. We calculated the mean and median for these spaces for the *C. briggsae* and *C. elegans* genomes. We also extracted the coordinates of each protein-coding genome from our *C. remanei* assembly, and excluded genes that have not been placed on the genetic map. Our *C. remanei* dataset therefore includes roughly half of our assembled sequence. We calculated the intergenic space for our assembly as the length between protein-coding genes, and calculated the mean and median for this distribution (Table 2).

Gene-specific loss within self-fertilizing speices

We characterized individual genes that were lost in the self-fertile hermaphrodites by analyzing putative protein domains for *C. remanei* genes with orthologous counterparts in the outcrossing

C. japonica, *C. brenneri*, and *C. sinica* and no identifiable orthologous counterparts in the self-fertile *C. elegans*, *C. briggsae*, and *C. tropicalis*. *Caenorhabditis* genomes have large numbers of nematode-specific and species-specific proteins [46], and high divergence makes it difficult to conclusively identify individual genes that are present in outcrossing *Caenorhabditis* but lost or nonfunctional in self-fertile species. Analyzing functional divergence is also complicated by the fact that functional information on nematode proteins comes largely from *C. elegans* studies and there is little information available for proteins that are not important to *C. elegans* biology. For example, although the outcrossing *Caenorhabditis* have gene complements 10-40% larger than the self-fertile *Caenorhabditis* we were only able to identify 11 *C. remanei* genes with orthologous counterparts in the outcrossing *Caenorhabditis* and no identifiable orthologous counterparts in the self-fertile *Caenorhabditis*. Five of these genes have no functional annotations, one contains a sterile alpha motif/pointed domain (IPR001660/IPR013761) common in protein-protein interactions, one contains a DDE superfamily endonuclease domain (PF13358) similar to the Tc1 and Tc3 tranposases in *C. elegans* and the Tcb1 and Tcb2 transposases in *C. briggsae*, and four contain CAP domains (IPR014044).

Cysteine-rich secretory proteins, antigen 5, and pathogenesis-related 1 (CAP) superfamily proteins have an array of functions including spermatogenesis, epididymal maturation, sperm capacitation and sperm-egg fusion in mammals, and immune regulation in invertebrates and plants [S1]. The CAP superfamily appears to have undergone species-specific evolution in the self-fertile *C. elegans* and *C. briggsae* and >50% of CAP proteins in each of these species have no orthologous counterparts in other *Caenorhabditis* (S4 Table). In contrast to this, >70% of CAP proteins in *C. remanei* have orthologous counterparts in at least one other *Caenorhabditis* species. It is not known if CAP proteins function in male reproduction or immune function in *Caenorhabditis*, and further functional studies are necessary to understand why CAP proteins may play different roles in outcrossing and selfing *Caenorhabditis*.

C. japonica is the most distantly related member of the Elegans supergroup and an outgroup for the more closely related Elegans group [13]. When we exclude *C. japonica*, we find 198 *C. remanei* genes with orthologous counterparts in the outcrossing *C. brenneri* and *C. sinica* and no orthologous counterparts in the self-fertile *Caenorhabditis*. Among these orthologous genes the most common functional annotations are: 11 proteins with F-box domains (IPR001810); 10 proteins with CAP domains (IPR014044); 7 proteins of unknown function DUF3557 (IPR021942); and 6 Argonaute/Dicer PAZ domains (IPR003100; S4 Table). The function of the PAZ (Piwi Argonaut and Zwille) domain is not known but it is found in members of the Piwi and Dicer families and thought to contribute to post-transcriptional gene regulation [S2,S3,S4,S5]. There is little information on the evolution of gene regulation between outcrossing and selfing *Caenorhabditis* and the specifics of these differences awaits more detailed analysis.

Comparative analysis of molecular pathways

We characterized the putative molecular pathways and interactomes for proteins for the 50 most over- and under-enriched in *C. remanei* relative to *C. elegans* across the entire group of genomes analyzed here (S3 Figure). Each value is scaled relative to the top pathway identified in each species, and pathways are grouped by biological function. Overall, these species have different numbers of proteins involved in signaling, neural development, cellular processes and multiple types of metabolism. Relative to *C. elegans*, the *C. remanei* genome is enriched for proteins

implicated in nucleotide metabolism, as well as those involved in amino acid, lipid and other types of metabolism, and under-enriched for proteins involved in carbohydrate metabolism and neural development. Overall, however, like the protein domains analyzed in the full paper, all species show similar distributions of pathway utilization.

Supplementary References

[S1] Gibbs GM, Roelants K, O'Bryan MK (2008) The cap superfamily: cystine-rich secretory proteins, antigen5, and pathogensis-related 1 proteins– roles in reproduction, cancer, and immune defense. Endocrine Reviews 29: 865-897.

[S2] Cerutti L, Mian N, Bateman A (2000) Domains in gene silencing and cell differentiation proteins: the novel PAZ domain and redefinition of the Piwi domain. Trends in Biochemical Sciences 25: 481-482.

[S3] Lingel A, Simon B, Izaurralde E, Sattler M (2003) Structure and nucleic-acid binding of the *Drosophila* Argonaute 2 PAZ domain. Nature 426: 465-468.

[S4] Song JJ, Liu J, Tolia NH, Schneiderman J, Smith SK, et al. (2003) The crystal structure of the argonaute2 PAZ domain reveals an RNA binding motif in RNAi effector complexes. Nature Structural Biology 10: 1026-1032.

[S5] Yan KS, Yan S, Farooq A, Han A, Zeng L, et al. (2003) Structure and conserved RNA binding of the PAZ domain. Nature: 468-474.
