# Supplementary figures and images for: Reproductive Mode and the Evolution of Genome Size and Structure in Caenorhabditis Nematodes
Source: PLoS Genet. 2015 Jun 26;11(6):e1005323. doi: 10.1371/journal.pgen.1005323 (PMC4482642; doi:10.1371/journal.pgen.1005323)

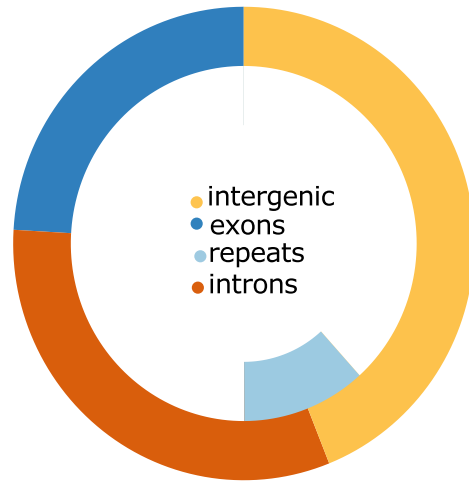

**S1 Figure.** Analysis of the Wormbase *C. remanei* genome sequence.

Supplement: S1 Fig — (PDF) [file pgen.1005323.s002.pdf]

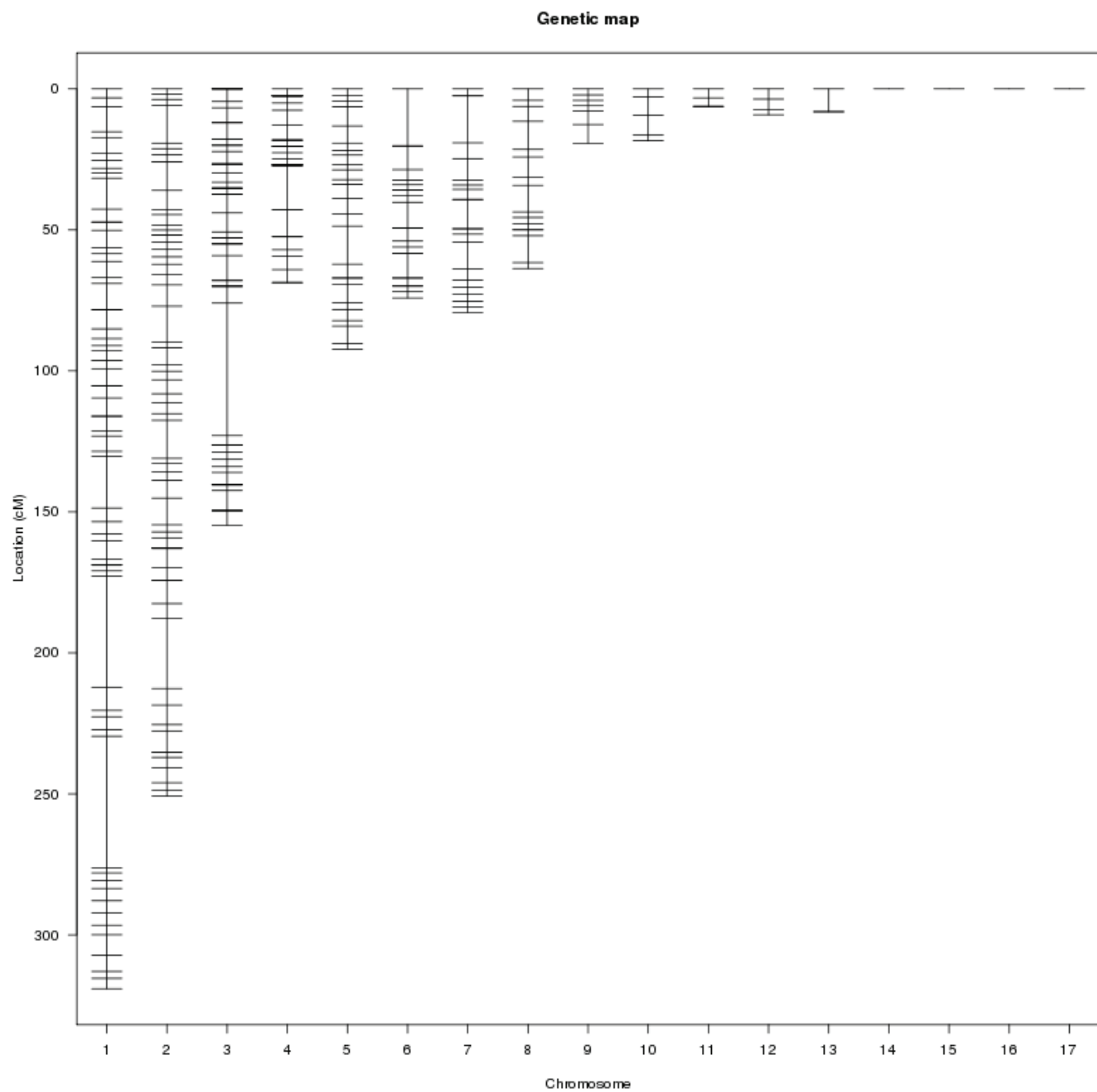

**S7 Figure.** *C. remanei* genetic map. Linkage groups plotted in centimorgans (cm).

Supplement: S7 Fig — (PDF) [file pgen.1005323.s008.pdf]

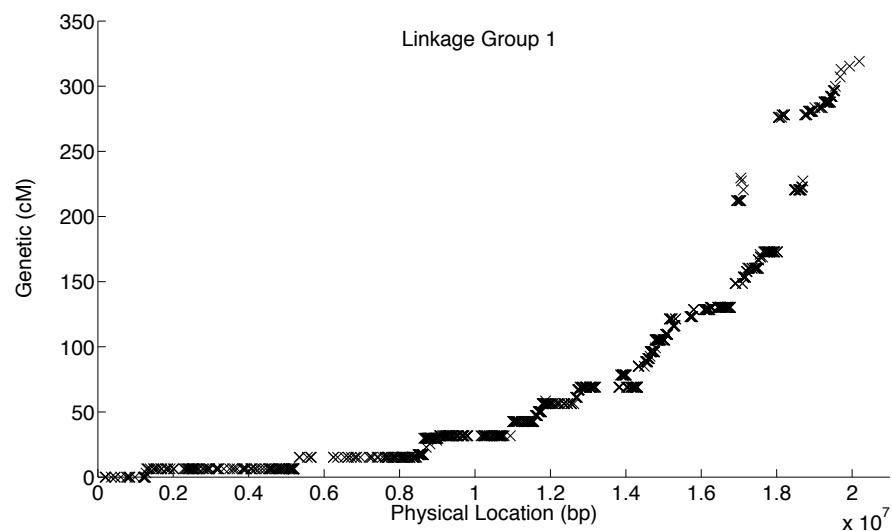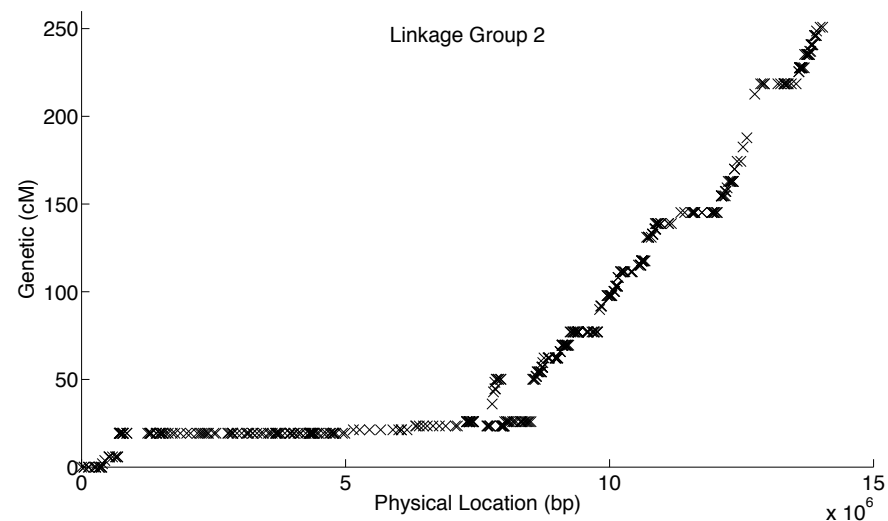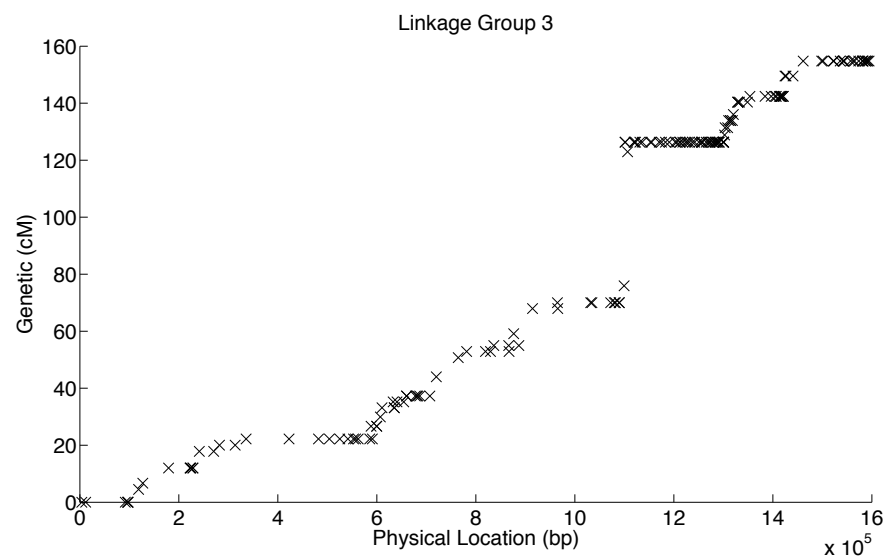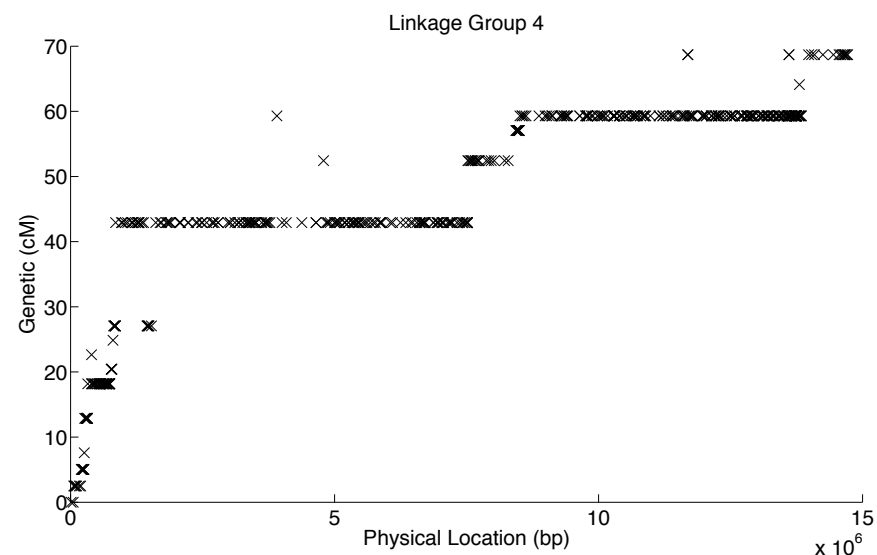

**S8 Figure.** Relationship between physical and genetic maps for linkage groups 1-4.

Supplement: S8 Fig — (PDF) [file pgen.1005323.s009.pdf]

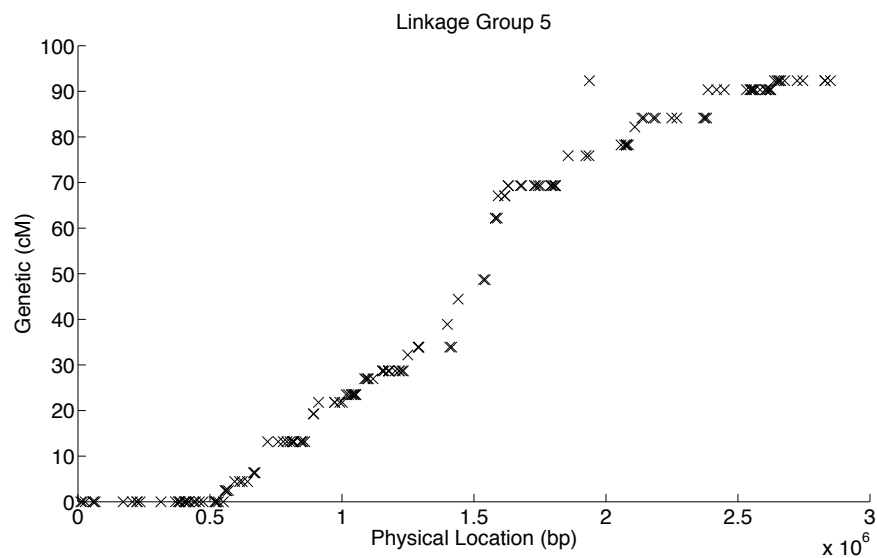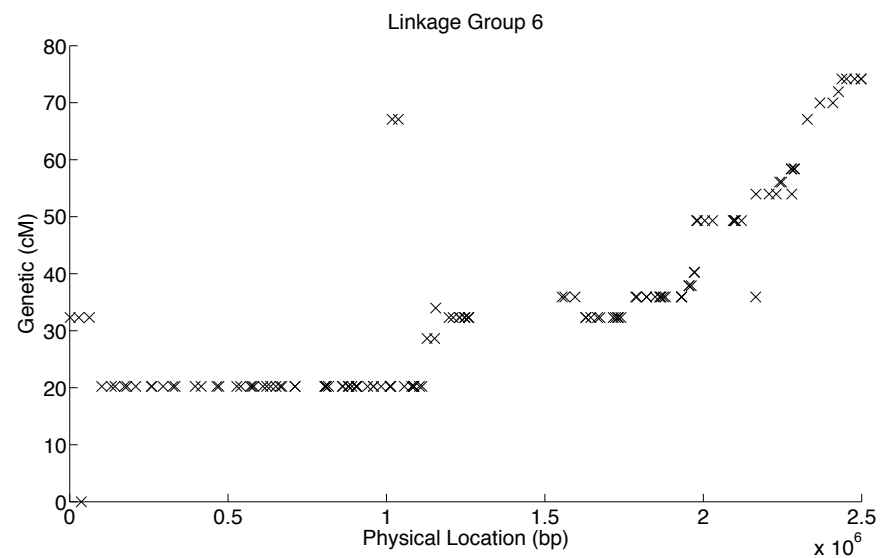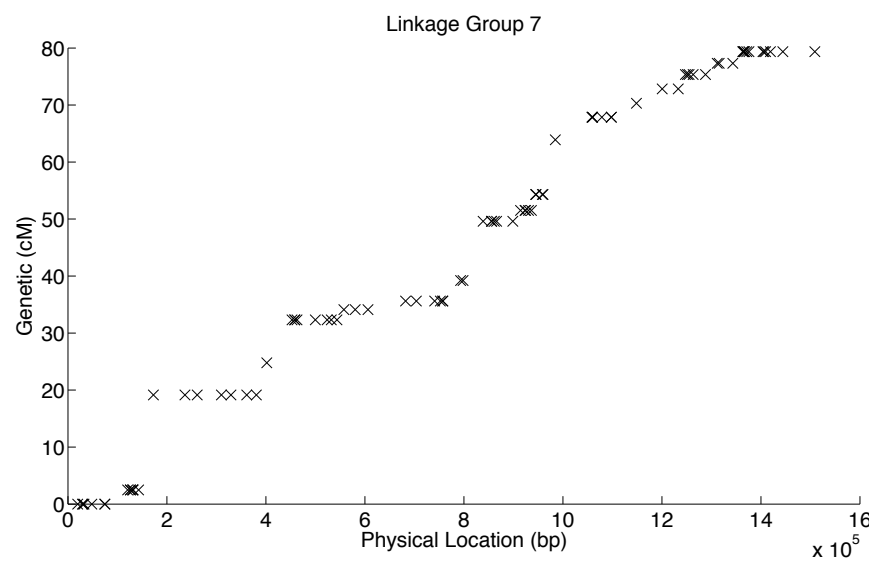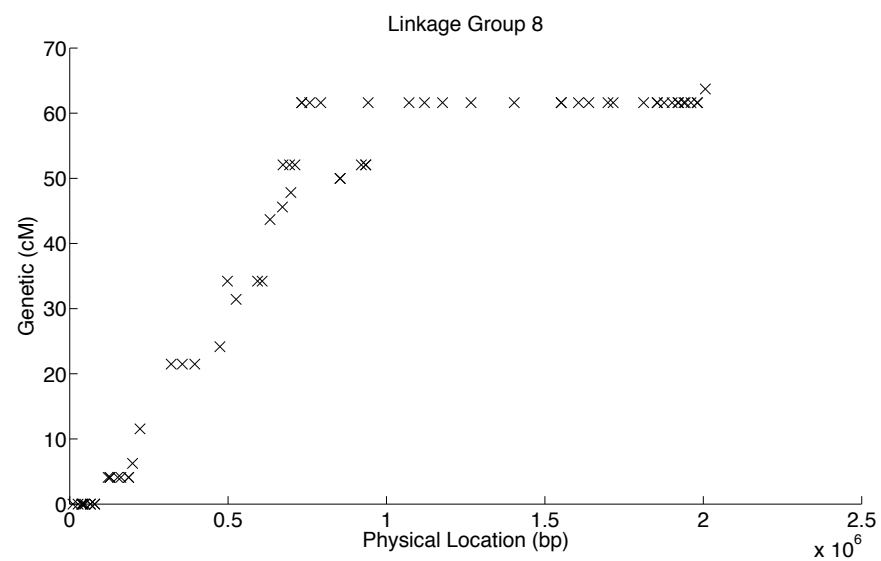

**S9 Figure.** Relationship between physical and genetic maps for linkage groups 5-8.

Supplement: S9 Fig — (PDF) [file pgen.1005323.s010.pdf]

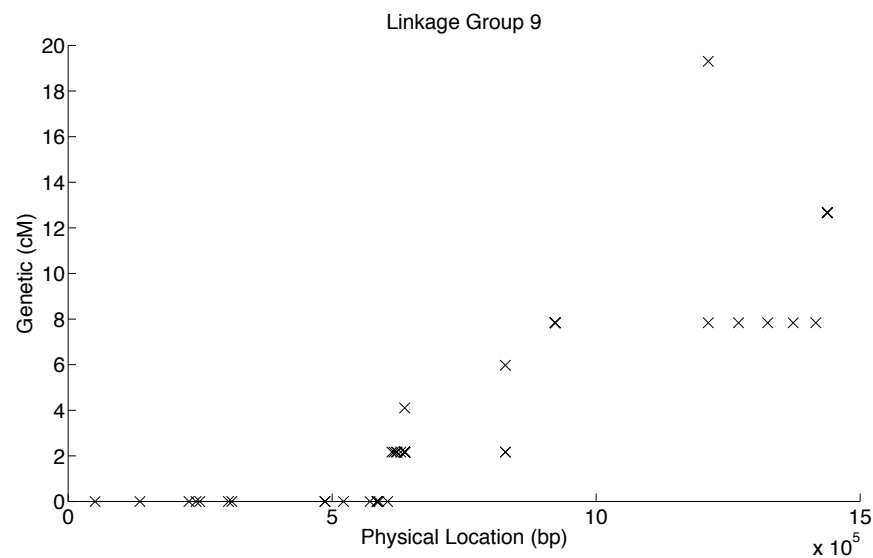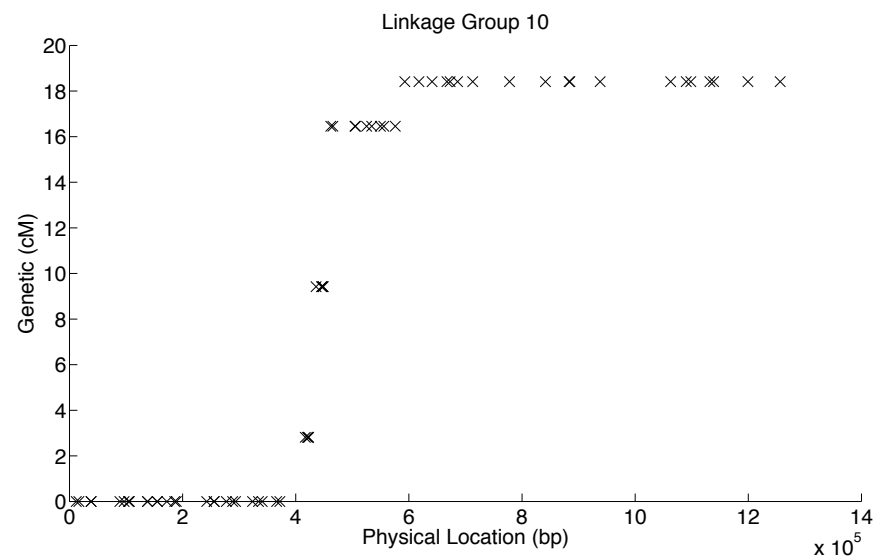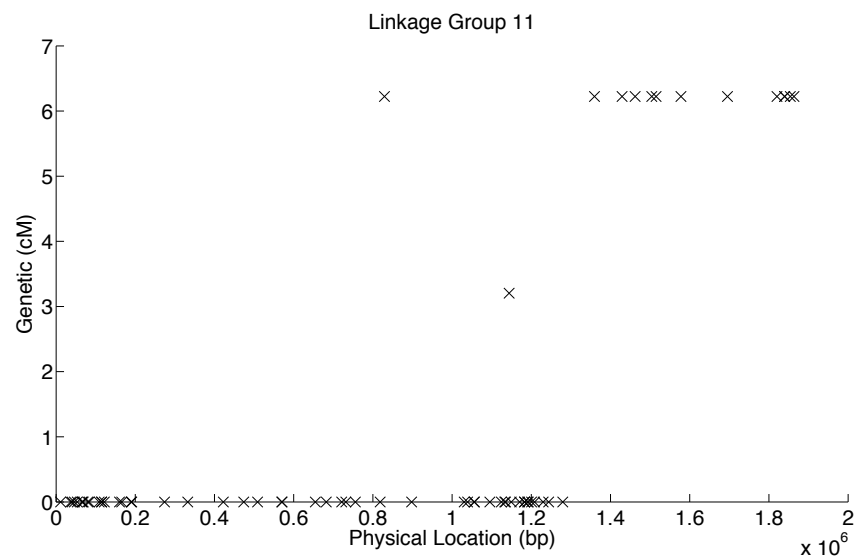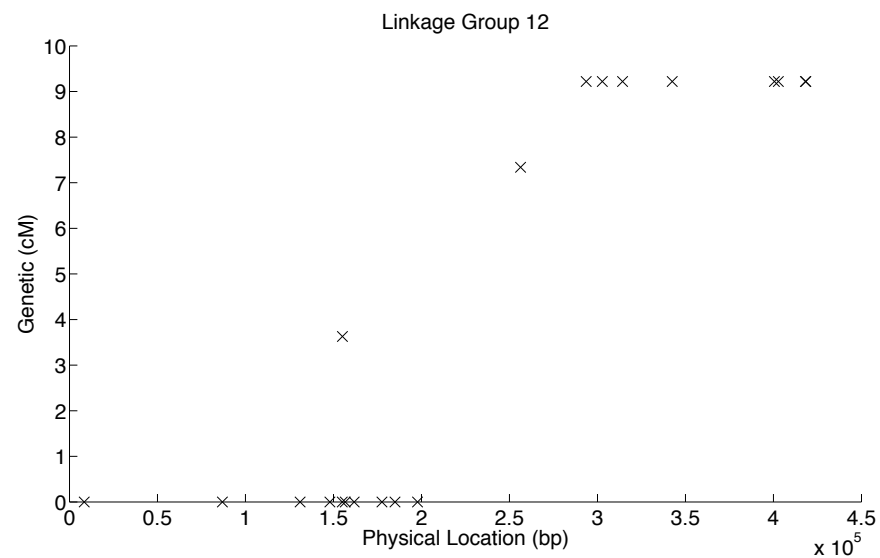

**S10 Figure.** Relationship between physical and genetic maps for linkage groups 9-12.

Supplement: S10 Fig — (PDF) [file pgen.1005323.s011.pdf]

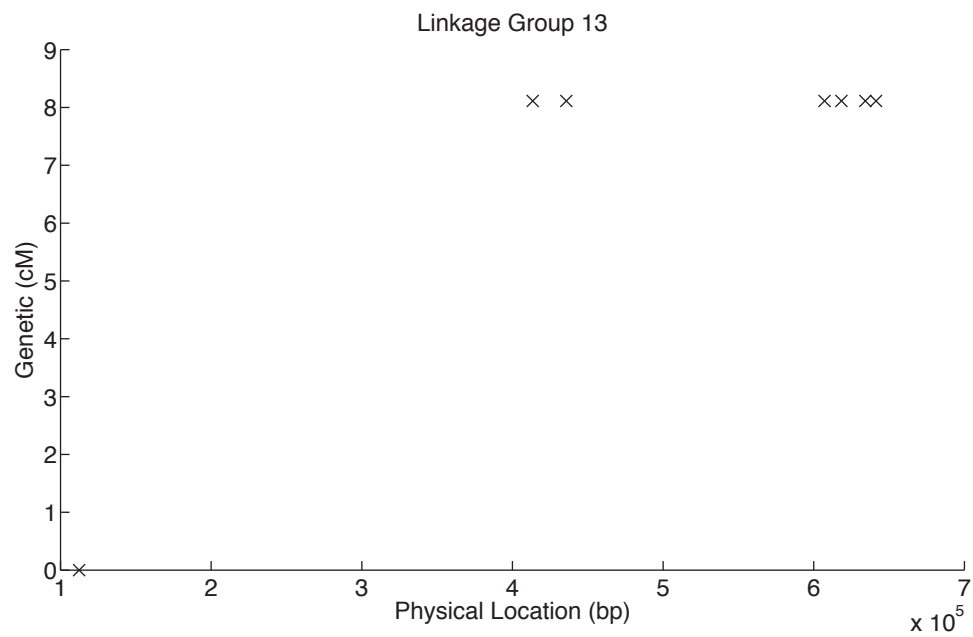

**S11 Figure.** Relationship between physical and genetic maps for linkage groups 13.

Supplement: S11 Fig — (PDF) [file pgen.1005323.s012.pdf]
