# Supplementary material for: Reproductive Mode and the Evolution of Genome Size and Structure in Caenorhabditis Nematodes
Source: PLoS Genet. 2015 Jun 26;11(6):e1005323. doi: 10.1371/journal.pgen.1005323 (PMC4482642; doi:10.1371/journal.pgen.1005323)
Supplement: S2 Fig — (PDF) [file pgen.1005323.s003.pdf]

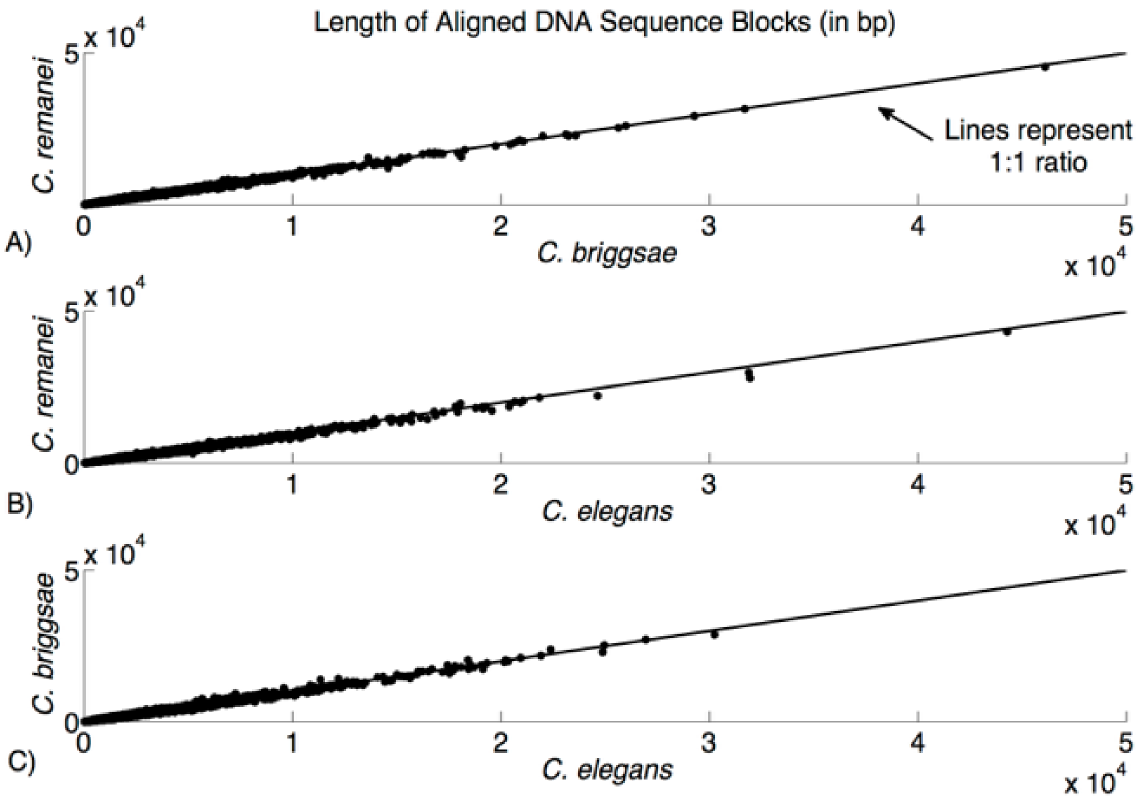

S2 Figure. Aligned blocks of sequence between (A) *C. remanei* and *C. briggsae*, (B) *C. remanei* and *C. elegans*, and (C) *C. briggsae* and *C. elegans* do not show size bias.
