# Supplementary material for: Reproductive Mode and the Evolution of Genome Size and Structure in Caenorhabditis Nematodes
Source: PLoS Genet. 2015 Jun 26;11(6):e1005323. doi: 10.1371/journal.pgen.1005323 (PMC4482642; doi:10.1371/journal.pgen.1005323)
Supplement: S3 Fig — Each value is scaled relative to the top pathway identified in each species, and pathways are grouped by biological function. C. remanei has a significant overrepresentation of pathway components involved in cellular processes, nucleotide metabolism, lipid metabolism and amino acid metabolism and a significant underrepresentation of pathway components involved in neural development and carbohydrate metabolism. (PDF) [file pgen.1005323.s004.pdf]

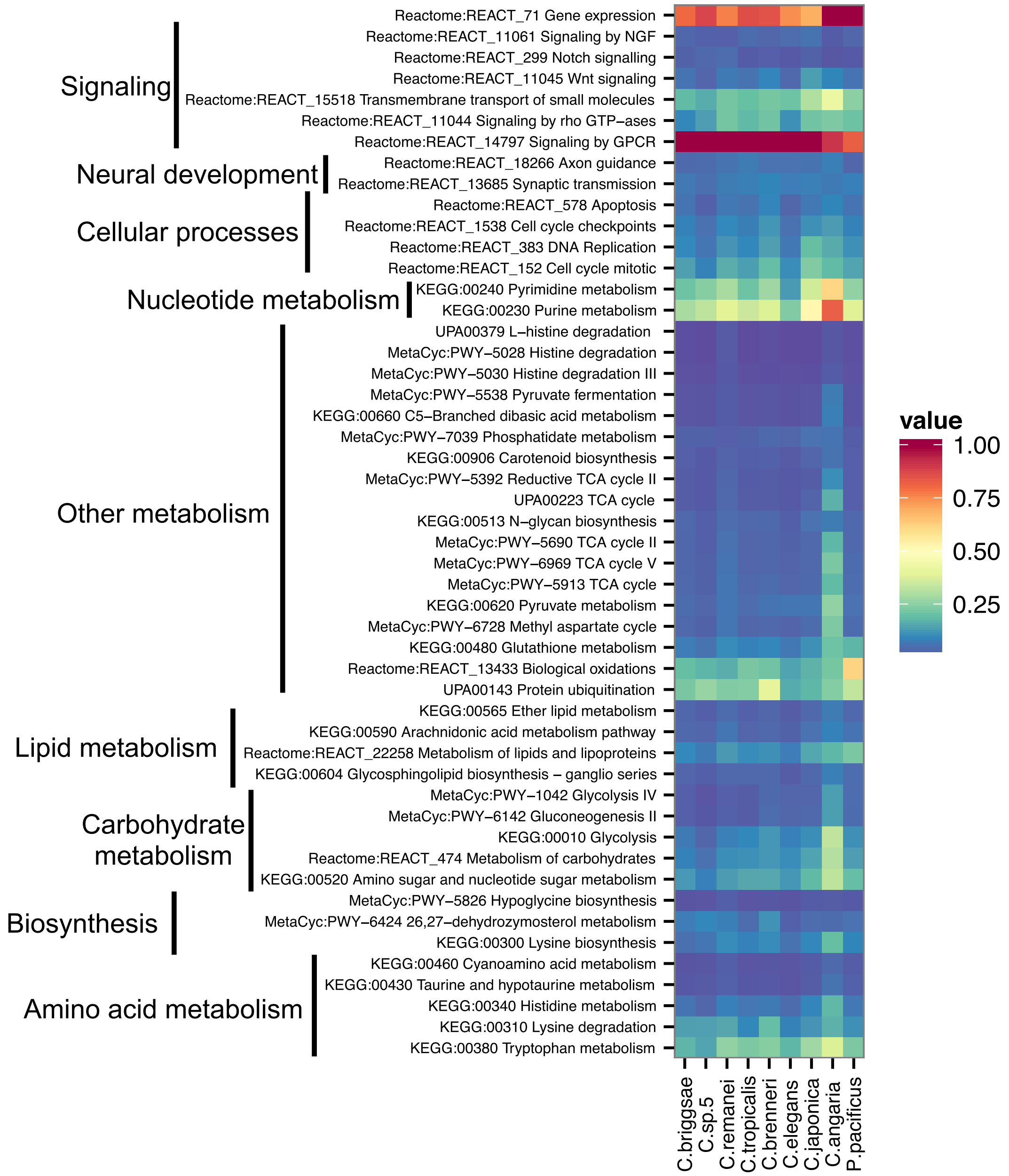

S3 Figure. The 50 most significantly over- and under-enriched pathway components ($p < 0.001$) in the *C. remanei* genome (as compared to the *C. elegans* genome. Each value is scaled relative to the top pathway identified in each species, and pathways are grouped by biological function. *C. remanei* has a significant overrepresentation of pathway components involved in cellular processes, nucleotide metabolism, lipid metabolism and amino acid metabolism and a significant underrepresentation of pathway components involved in neural development and carbohydrate metabolism.
