# Supplementary material for: Reproductive Mode and the Evolution of Genome Size and Structure in Caenorhabditis Nematodes
Source: PLoS Genet. 2015 Jun 26;11(6):e1005323. doi: 10.1371/journal.pgen.1005323 (PMC4482642; doi:10.1371/journal.pgen.1005323)
Supplement: S4 Fig — A) The training data used for the decision tree. B) Initial assignments identified scaffolds of Caenorhabditis or non-Caenorhabditis origin. C) 1589 scaffolds were identified as either Caenorhabditis or non-Caenorhabditis with high probability and 11 scaffolds had ambiguous origins (pictured in green above). For the final set of C. remanei DNA we included all scaffolds with p>0.2. There are three scaffolds that BLAST identified as of Caenorhabditis origin but the GC/coverage profile indicated non-Caenorhabditis origin. We included these in the final assembly as well for the sake of completeness. (PDF) [file pgen.1005323.s005.pdf]

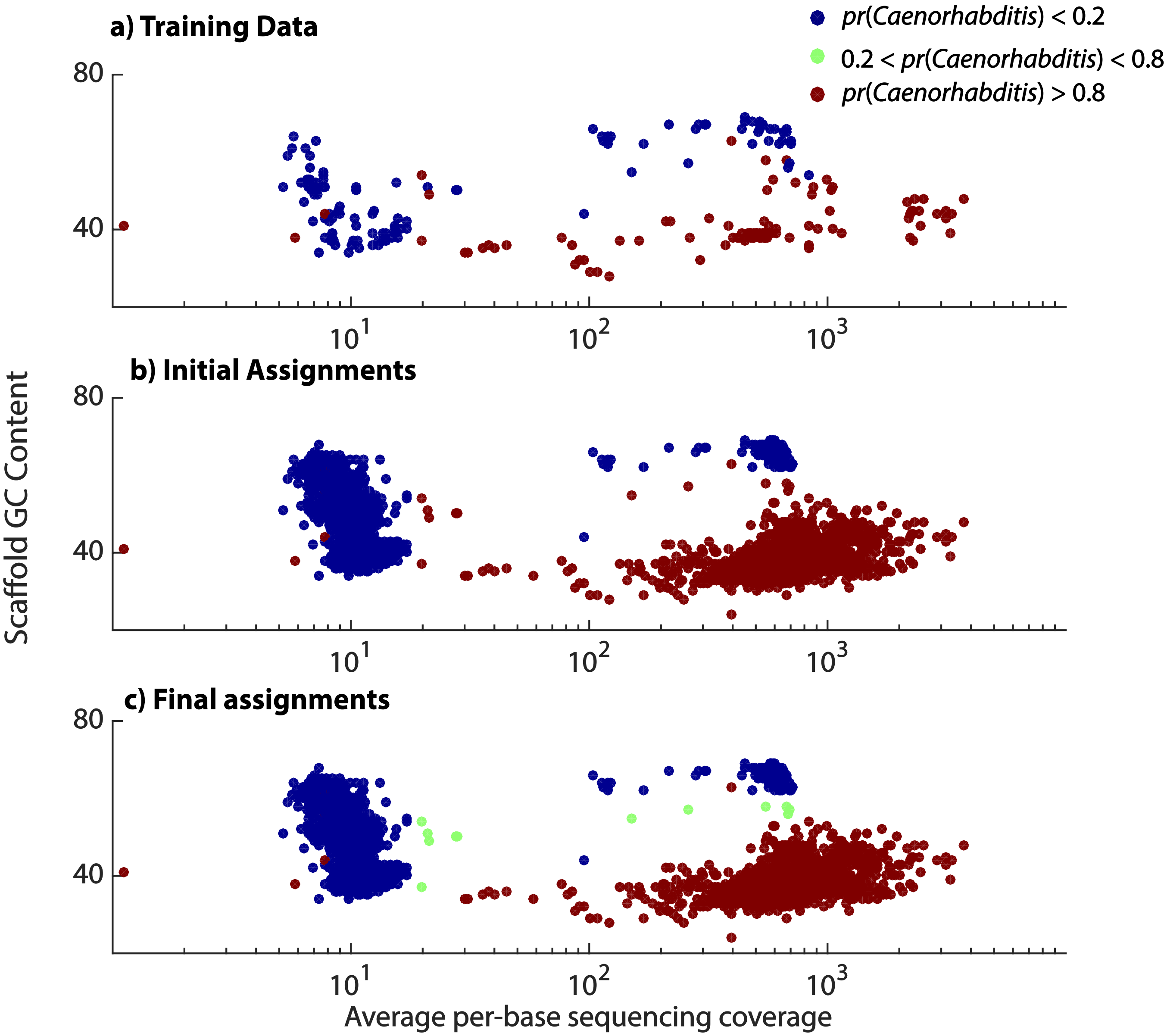

S4 Figure. Individual scaffolds were assigned *Caenorhabditis* or non-*Caenorhabditis* origin based on GC content and average per-base sequencing coverage. A) The training data used for the decision tree. B) Initial assignments identified scaffolds of *Caenorhabditis* or non-*Caenorhabditis* origin. C) 1589 scaffolds were identified as either *Caenorhabditis* or non-*Caenorhabditis* with high probability and 11 scaffolds had ambiguous origins (pictured in green above). For the final set of *C. remanei* DNA we included all scaffolds with $p > 0.2$. There are three scaffolds that BLAST identified as of *Caenorhabditis* origin but the GC/coverage profile indicated non-*Caenorhabditis* origin. We included these in the final assembly as well for the sake of completeness.
