# Supplementary material for: Reproductive Mode and the Evolution of Genome Size and Structure in Caenorhabditis Nematodes
Source: PLoS Genet. 2015 Jun 26;11(6):e1005323. doi: 10.1371/journal.pgen.1005323 (PMC4482642; doi:10.1371/journal.pgen.1005323)
Supplement: S5 Fig — Markers are plotted along each scaffold and scaffolds are ordered by length. (PDF) [file pgen.1005323.s006.pdf]

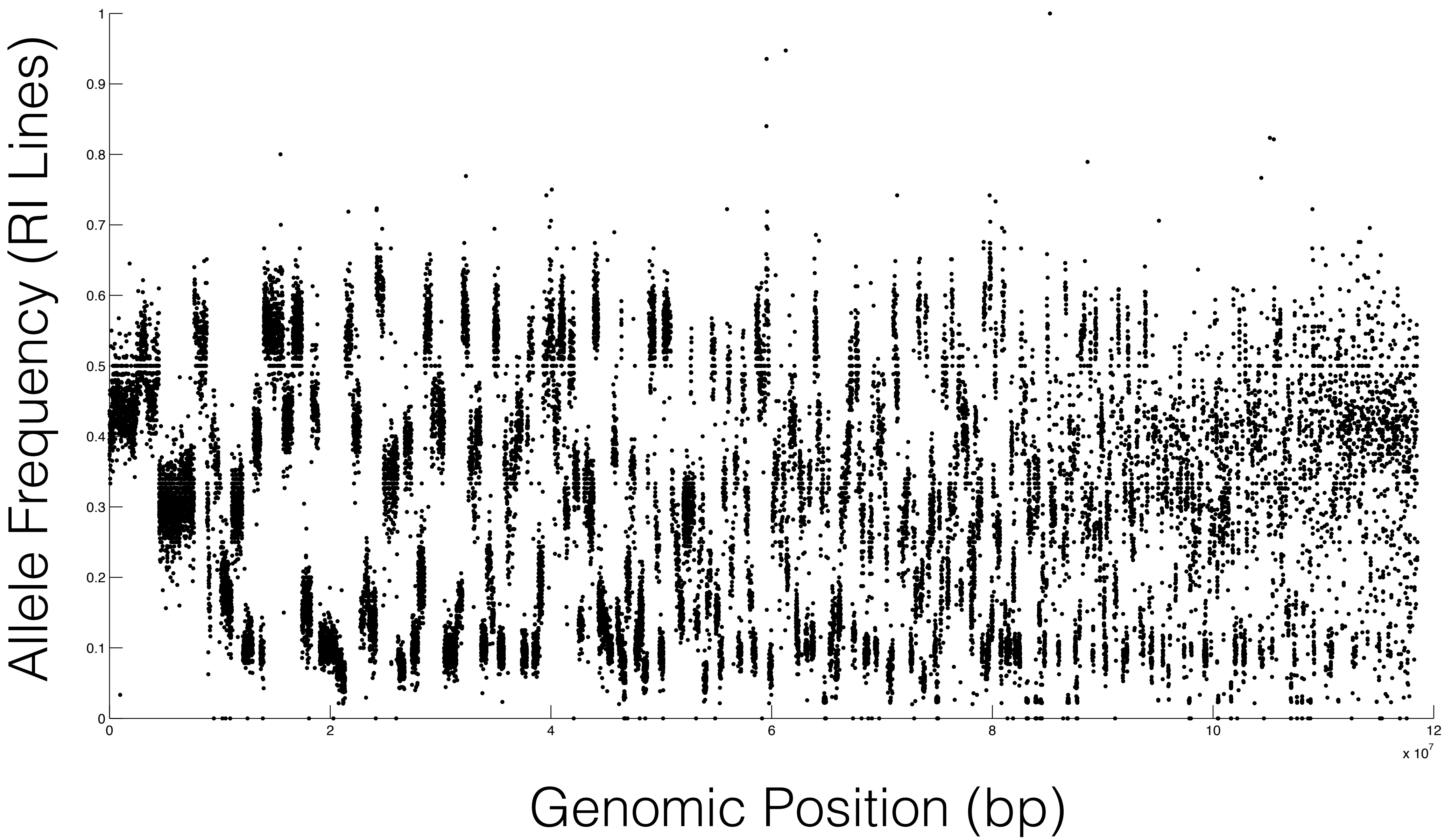

S5 Figure. Allele frequencies for every RAD-associated SNP marker.} Markers are plotted along each scaffold and scaffolds are ordered by length.
