# Supplementary material for: Reproductive Mode and the Evolution of Genome Size and Structure in Caenorhabditis Nematodes
Source: PLoS Genet. 2015 Jun 26;11(6):e1005323. doi: 10.1371/journal.pgen.1005323 (PMC4482642; doi:10.1371/journal.pgen.1005323)
Supplement: S6 Fig — (PDF) [file pgen.1005323.s007.pdf]

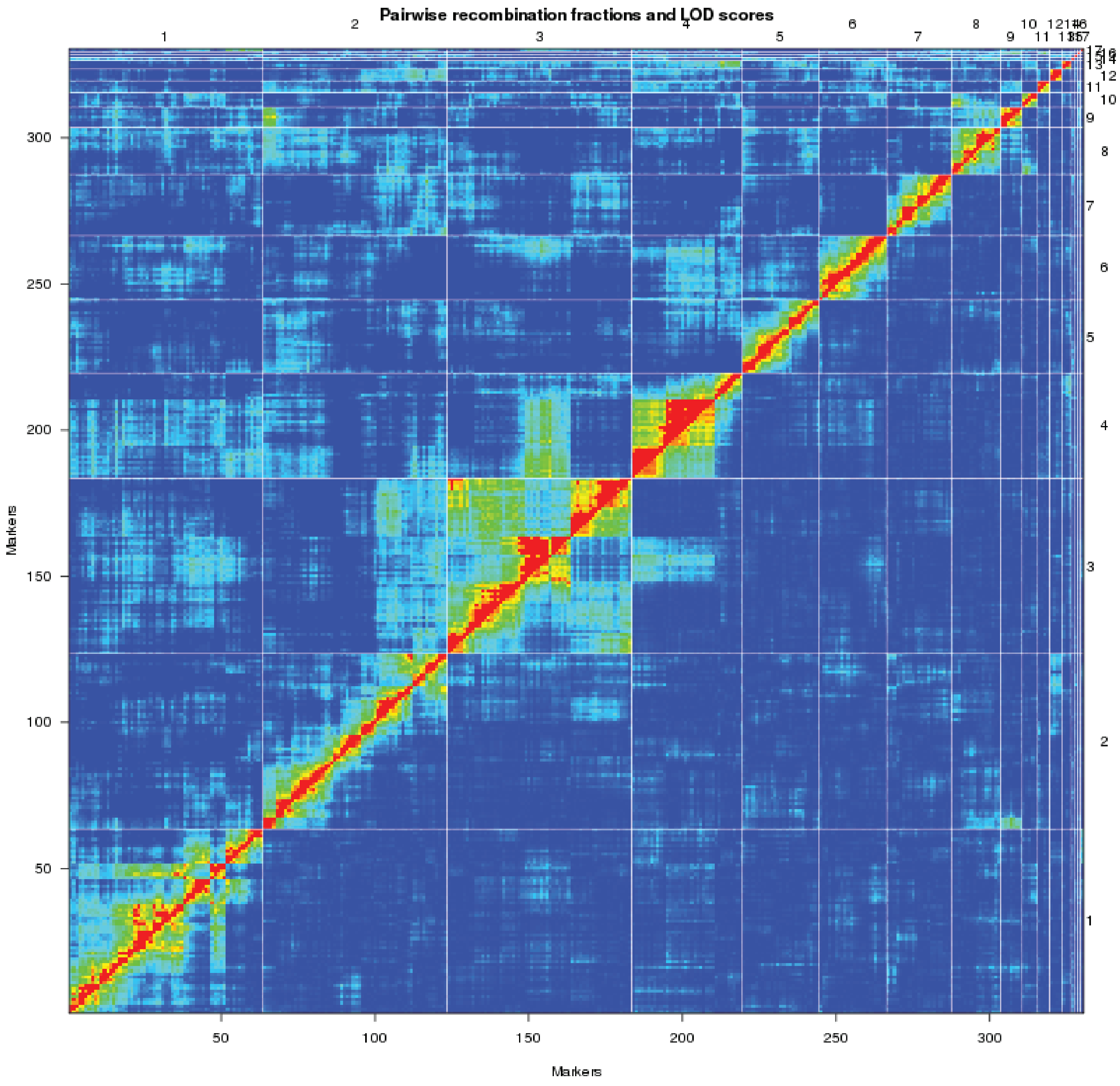

S6 Figure. Pairwise recombination fractions and LOD scores for the final genetic map. Areas of low recombination are shown in red and areas of high recombination are shown in blue.
