# Supplementary material for: Reproductive Mode and the Evolution of Genome Size and Structure in Caenorhabditis Nematodes
Source: PLoS Genet. 2015 Jun 26;11(6):e1005323. doi: 10.1371/journal.pgen.1005323 (PMC4482642; doi:10.1371/journal.pgen.1005323)
Supplement: S12 Fig — Roughly 50% of the 118.5Mb assembly is contained in 10 large linkage groups and scaffolds, and 90% of the length is contained in 160 linkage groups and scaffolds. (PDF) [file pgen.1005323.s013.pdf]

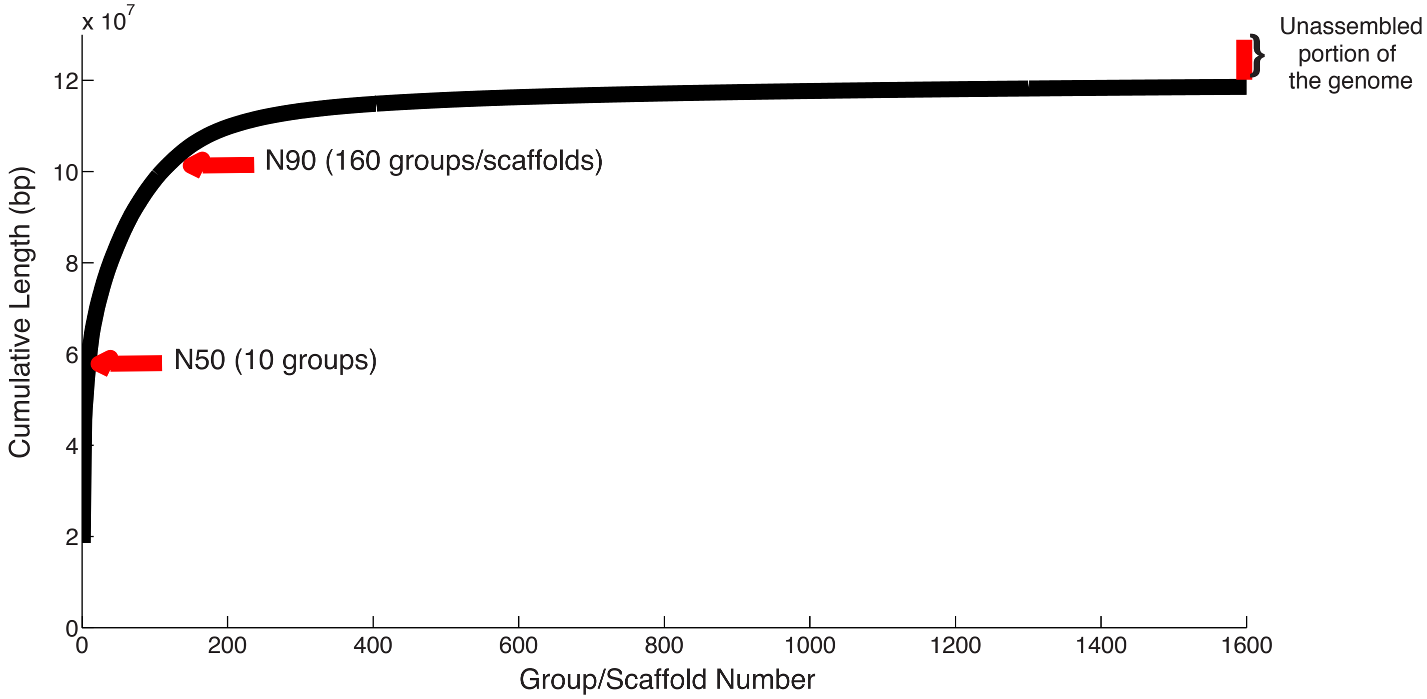

S12 Figure. The cumulative length distribution of the linkage groups and scaffolds. Roughly 50% of the 118.5Mb assembly is contained in 10 large linkage groups and scaffolds, and 90% of the length is contained in 160 linkage groups and scaffolds.
