# Supplementary material for: Reproductive Mode and the Evolution of Genome Size and Structure in Caenorhabditis Nematodes
Source: PLoS Genet. 2015 Jun 26;11(6):e1005323. doi: 10.1371/journal.pgen.1005323 (PMC4482642; doi:10.1371/journal.pgen.1005323)
Supplement: S1 Table — (PDF) [file pgen.1005323.s014.pdf]

S1 Table. Repetitive content and length aligned to the existing Wormbase *C. remanei* genome

Assembly	% Genome estimated repetitive	Length aligned
PX356 (1/2 data)	20	108.62
PX356 (full dataset)	22	106.64
1/2 data, haploidify	22	109.41
full data, haploidify option	20	104.69
full data, k-mer filtered (k=15)	16	110.01
full data, haploidify, k=11	10	89.63
full data, haploidify, k=13	12	96.35
**full data, haploidify, k=15**	**15**	**119.72**
full data, haploidify, k=17	15	114
full data, haploidify, filtered mate pairs	15	98.66
