# Supplementary material for: Reproductive Mode and the Evolution of Genome Size and Structure in Caenorhabditis Nematodes
Source: PLoS Genet. 2015 Jun 26;11(6):e1005323. doi: 10.1371/journal.pgen.1005323 (PMC4482642; doi:10.1371/journal.pgen.1005323)
Supplement: S2 Table — (PDF) [file pgen.1005323.s015.pdf]

S2 Table. Summary of genome statistics for each species as analyzed using our uniform pipeline.

	Self-reproducing (androdioecious)			Outcrossing (gonochoristic/dioecious)			
	C. elegans	*C. briggsae*	*C. tropicalis*	*C. sinica*	*C. brenneri*	*C. japonica*	*C. angaria*
**Size of assembled genome (Mb)**[1]							
Exons[2]	29	25	25	39	43	24	22
Introns[2]	35	33	16	29	45	41	25
Intergenic	37	50	38	63	103[3]	101	59
Total	100	108	79	131	190	166	105
**Repetitive content**							
Size (Mb)	13	24	6	18	29	70[4]	11
Proportion of the assembled genome	13%	22%	8%	14%	15%	42%	11%

[1]Reported values are for our re-annotation of these genomes using a uniform pipeline.

[2]Exonic, intronic, and intergenic portions can sum to greater than the total genome size because of alternative splicing. The exonic portion is underestimated by our computational pipeline because we are predicting fewer alternative mRNA models than have been experimentally validated.

[3]Our re-annotation predicted a much larger genic footprint for *C. brenneri* and a much larger repeat content for *C. japonica*, consistent with previous findings on allelism in both assemblies [19].
