# Supplementary material for: Reproductive Mode and the Evolution of Genome Size and Structure in Caenorhabditis Nematodes
Source: PLoS Genet. 2015 Jun 26;11(6):e1005323. doi: 10.1371/journal.pgen.1005323 (PMC4482642; doi:10.1371/journal.pgen.1005323)
Supplement: S3 Table — (PDF) [file pgen.1005323.s016.pdf]

S3 Table. Assembly size, contig length, scaffold length, N50 for contigs and N50 for scaffolds. All lengths are given in Mb. The final parameter choices are shown in bold in the table.

Assembly	Contig Length	Scaffold Length	N50 Contig	N50 Scaffold
PX356 (1/2 data)	125.82	131.06	41.5	305
PX356 (full dataset)	133.05	139.06	23.4	74
1/2 data, haploidify	125.75	130.7	42.4	313
full data, haploidify option	131.49	138.3	25.9	112
full data, k-mer filtered (k=15)	134.69	139.58	24.5	59
full data, haploidify, k=11	111.66	113.7	5.4	6
full data, haploidify, k=13	122.4	132.8	17.1	142
**full data, haploidify, k=15**	**130.52**	**136.79**	**34.9**	**311**
full data, haploidify, k=17	113.6	114	8	12
full data, haploidify, filtered mate pairs	126.4	132.52	25.5	252
