# Supplementary material for: Reproductive Mode and the Evolution of Genome Size and Structure in Caenorhabditis Nematodes
Source: PLoS Genet. 2015 Jun 26;11(6):e1005323. doi: 10.1371/journal.pgen.1005323 (PMC4482642; doi:10.1371/journal.pgen.1005323)
Supplement: S4 Table — (PDF) [file pgen.1005323.s017.pdf]

S4 Table. Interproscan protein annotation categories for proteins found in outcrossing *Caenorhabditis* (Elegans supergroup: *C. sinica, C. brenneri,* and *C. remanei*) but absent in the self-fertile *Caenorhabditis.*

Number Annotated	IPR Category
11	IPR001810 F-box domain
10	IPR014044 CAP domain
7	IPR021942 Protein of unknown function DUF3557
6	IPR003100 Argonaute/Dicer protein, PAZ domain
6	IPR002900 Domain of unknown function DUF38, Caenorhabditis species
5	IPR027417 P-loop containing nucleoside triphosphate hydrolase
5	IPR012885 F-box associated domain, type 2
5	IPR011333 BTB/POZ fold
4	IPR013083 Zinc finger, RING/FYVE/PHD-type
4	IPR012337 Ribonuclease H-like domain
4	IPR001841 Zinc finger, RING-type
4	IPR000210 BTB/POZ-like
3	IPR018817 7TM GPCR, serpentine receptor class z (Srz)
3	IPR017907 Zinc finger, RING-type, conserved site
3	IPR016187 C-type lectin fold
3	IPR016186 C-type lectin-like
3	IPR013069 BTB/POZ
3	IPR006186 Serine/threonine-specific protein phosphatase
3	IPR004843 Phosphoesterase domain
3	IPR001584 Integrase, catalytic core
3	IPR001304 C-type lectin
2	IPR027785 UvrD-like helicase C-terminal domain
2	IPR024079 Metallopeptidase, catalytic domain
2	IPR023780 Chromo domain
2	IPR019427 7TM GPCR, serpentine receptor class w (Srw)
2	IPR017853 Glycoside hydrolase, superfamily
2	IPR017452 GPCR, rhodopsin-like, 7TM
2	IPR016197 Chromo domain-like
2	IPR013781 Glycoside hydrolase, catalytic domain
2	IPR013088 Zinc finger, NHR/GATA-type
2	IPR011583 Chitinase II
2	IPR011009 Protein kinase-like domain
2	IPR010285 DNA helicase Pif1 like
2	IPR008974 TRAF-like
2	IPR008946 Nuclear hormone receptor, ligand-binding
2	IPR008271 Serine/threonine-protein kinase, active site
2	IPR005312 Protein of unknown function DUF1759
2	IPR004875 DDE superfamily endonuclease, CENP-B-like
2	IPR003839 7TM GPCR, serpentine receptor class u (Sru)
2	IPR003131 Potassium channel tetramerisation-type BTB domain
2	IPR003125 Domain of unknown function WSN
2	IPR002290 Serine/threonine- /dual specificity protein kinase, catalytic domain
2	IPR002083 MATH

S3 Table continued

Number Annotated	IPR Category
2	IPR001628 Zinc finger, nuclear hormone receptor-type
2	IPR001223 Glycoside hydrolase, family 18, catalytic domain
2	IPR000953 Chromo domain/shadow
2	IPR000719 Protein kinase domain
2	IPR000609 7TM GPCR, serpentine receptor class g (Srg)
2	IPR000536 Nuclear hormone receptor, ligand-binding, core
2	IPR000494 EGF receptor, L domain
2	IPR000242 Protein-tyrosine phosphatase, receptor/non-receptor type
1	IPR023631 Amidase signature domain
1	IPR021129 Sterile alpha motif, type 1
1	IPR021109 Aspartic peptidase domain
1	IPR020904 Short-chain dehydrogenase/reductase, conserved site
1	IPR019430 7TM GPCR, serpentine receptor class x (Srx)
1	IPR019428 7TM GPCR, serpentine receptor class r (Str)
1	IPR019425 7TM GPCR, serpentine receptor class t (Srt)
1	IPR019422 7TM GPCR, serpentine receptor class h (Srh)
1	IPR019421 7TM GPCR, serpentine receptor class d (Srd)
1	IPR019332 Organic solute carrier protein 1
1	IPR018497 Peptidase M13, C-terminal domain
1	IPR018289 MULE transposase domain
1	IPR016090 Phospholipase A2 domain
1	IPR016040 NAD(P)-binding domain
1	IPR013783 Immunoglobulin-like fold
1	IPR013761 Sterile alpha motif/pointed domain
1	IPR013151 Immunoglobulin
1	IPR013098 Immunoglobulin I-set
1	IPR013087 Zinc finger C2H2-type/integrase DNA-binding domain
1	IPR012336 Thioredoxin-like fold
1	IPR009854 Orthoreovirus membrane fusion p10
1	IPR009564 Protein of unknown function DUF1179
1	IPR009057 Homeodomain-like
1	IPR008753 Peptidase M13, N-terminal domain
1	IPR007883 Protein of unknown function DUF713
1	IPR007110 Immunoglobulin-like domain
1	IPR007087 Zinc finger, C2H2
1	IPR006621 Nose resistant-to-fluoxetine protein, N-terminal
1	IPR006600 HTH CenpB-type DNA-binding domain
1	IPR006583 PAN-3 domain
1	IPR006026 Peptidase, metallopeptidase
1	IPR005514 Protein of unknown function DUF316
1	IPR004045 Glutathione S-transferase, N-terminal
1	IPR003677 Domain of unknown function DUF148

S3 Table continued

Number Annotated	IPR Category
1	IPR003599 Immunoglobulin subtype
1	IPR003598 Immunoglobulin subtype 2
1	IPR003392 Patched
1	IPR002656 Acyltransferase 3
1	IPR002516 Glycosyl transferase, family 11
1	IPR002347 Glucose/ribitol dehydrogenase
1	IPR002198 Short-chain dehydrogenase/reductase SDR
1	IPR002156 Ribonuclease H domain
1	IPR001781 Zinc finger, LIM-type
1	IPR001683 Phox homologous domain
1	IPR001660 Sterile alpha motif domain
1	IPR001534 Transthyretin-like
1	IPR001506 Peptidase M12A, astacin
1	IPR001461 Aspartic peptidase
1	IPR000731 Sterol-sensing domain
1	IPR000605 Helicase, superfamily 3, single-stranded DNA/RNA virus
1	IPR000542 Acyltransferase ChoActase/COT/CPT
1	IPR000276 G protein-coupled receptor, rhodopsin-like
1	IPR000120 Amidase
1	IPR000008 C2 domain
